# A novel, essential *trans*-splicing protein connects the nematode SL1 snRNP to the CBC-ARS2 complex

**DOI:** 10.1093/nar/gkac534

**Published:** 2022-06-23

**Authors:** Rotimi Yemi Fasimoye, Rosie Elizabeth Barker Spencer, Eva Soto-Martin, Peter Eijlers, Haitem Elmassoudi, Sarah Brivio, Carolina Mangana, Viktorija Sabele, Radoslava Rechtorikova, Marius Wenzel, Bernadette Connolly, Jonathan Pettitt, Berndt Müller

**Affiliations:** School of Medicine, Medical Sciences and Nutrition, University of Aberdeen, Institute of Medical Sciences, Foresterhill, Aberdeen AB25 2ZD, Scotland, UK; School of Medicine, Medical Sciences and Nutrition, University of Aberdeen, Institute of Medical Sciences, Foresterhill, Aberdeen AB25 2ZD, Scotland, UK; School of Medicine, Medical Sciences and Nutrition, University of Aberdeen, Institute of Medical Sciences, Foresterhill, Aberdeen AB25 2ZD, Scotland, UK; School of Medicine, Medical Sciences and Nutrition, University of Aberdeen, Institute of Medical Sciences, Foresterhill, Aberdeen AB25 2ZD, Scotland, UK; School of Medicine, Medical Sciences and Nutrition, University of Aberdeen, Institute of Medical Sciences, Foresterhill, Aberdeen AB25 2ZD, Scotland, UK; School of Medicine, Medical Sciences and Nutrition, University of Aberdeen, Institute of Medical Sciences, Foresterhill, Aberdeen AB25 2ZD, Scotland, UK; School of Medicine, Medical Sciences and Nutrition, University of Aberdeen, Institute of Medical Sciences, Foresterhill, Aberdeen AB25 2ZD, Scotland, UK; School of Medicine, Medical Sciences and Nutrition, University of Aberdeen, Institute of Medical Sciences, Foresterhill, Aberdeen AB25 2ZD, Scotland, UK; School of Medicine, Medical Sciences and Nutrition, University of Aberdeen, Institute of Medical Sciences, Foresterhill, Aberdeen AB25 2ZD, Scotland, UK; Centre of Genome-Enabled Biology and Medicine, University of Aberdeen, 23 St Machar Drive, Aberdeen AB24 3RY, Scotland, UK; School of Medicine, Medical Sciences and Nutrition, University of Aberdeen, Institute of Medical Sciences, Foresterhill, Aberdeen AB25 2ZD, Scotland, UK; School of Medicine, Medical Sciences and Nutrition, University of Aberdeen, Institute of Medical Sciences, Foresterhill, Aberdeen AB25 2ZD, Scotland, UK; School of Medicine, Medical Sciences and Nutrition, University of Aberdeen, Institute of Medical Sciences, Foresterhill, Aberdeen AB25 2ZD, Scotland, UK

## Abstract

Spliced leader *trans*-splicing is essential for gene expression in many eukaryotes. To elucidate the molecular mechanism of this process, we characterise the molecules associated with the *Caenorhabditis elegans* major spliced leader snRNP (SL1 snRNP), which donates the spliced leader that replaces the 5′ untranslated region of most pre-mRNAs. Using a GFP-tagged version of the SL1 snRNP protein SNA-1 created by CRISPR-mediated genome engineering, we immunoprecipitate and identify RNAs and protein components by RIP-Seq and mass spectrometry. This reveals the composition of the SL1 snRNP and identifies associations with spliceosome components PRP-8 and PRP-19. Significantly, we identify a novel, nematode-specific protein required for SL1 *trans*-splicing, which we designate SNA-3. SNA-3 is an essential, nuclear protein with three NADAR domains whose function is unknown. Mutation of key residues in NADAR domains inactivates the protein, indicating that domain function is required for activity. SNA-3 interacts with the CBC-ARS2 complex and other factors involved in RNA metabolism, including SUT-1 protein, through RNA or protein-mediated contacts revealed by yeast two-hybrid assays, localisation studies and immunoprecipitations. Our data are compatible with a role for SNA-3 in coordinating *trans*-splicing with target pre-mRNA transcription or in the processing of the Y-branch product of the *trans*-splicing reaction.

## INTRODUCTION

Spliced leader *trans*-splicing is an RNA processing step that occurs in many eukaryotes including kinetoplastids, nematodes, trematodes and tunicates (1). The *trans*-splicing reaction replaces the nascent 5′ end of pre-mRNAs with a trimethylguanosine*-*capped RNA sequence called the spliced leader (1), employing most of the same spliceosome components used in the removal of introns from the pre-mRNA. Spliced leader *trans*-splicing proceeds through two consecutive transesterification reactions that require the U2 and U4/U6.U5 snRNPs (2,3). The spliced leader is donated by a longer precursor, the spliced leader RNA (SL RNA), which provides the splice donor site. The 5′ end of the pre-mRNA, removed by *trans*-splicing, provides the splice acceptor site and is thus termed the outron.

The SL RNA forms a ribonucleoprotein complex (SL snRNP) with the Sm proteins that similarly interact with the U1, U2, U4 and U5 snRNAs of the spliceosome (4–6). The Sm proteins are the only conserved protein components known to be associated with SL RNAs in multiple eukaryotic groups. The SL RNAs of nematodes are known to interact with a pair of conserved, taxon-specific proteins, SNA-1 and SNA-2 (7,8), whose loss of function impairs spliced leader *trans*-splicing in *Caenorhabditis elegans* (9), but their precise functional roles have not been fully elucidated. Other factors implicated in nematode spliced leader *trans*-splicing include a paralogue of SNA-1, SUT-1 and a family of non-coding RNAs, the SmY RNAs (3,8), but even less is known about the roles played by these molecules.

Besides the spliced leader *trans*-spliced mRNA, the *trans*-splicing reaction produces a second product, the so-called ‘Y-branch’ RNA, the analogue of the lariat in *cis*-splicing. The fate of the Sm proteins associated with the Y-branch is unknown; they may be degraded or recycled, but the mechanism involved in either case remains to be determined.

In *C. elegans*, >85% of genes produce an mRNA that is *trans*-spliced to a spliced leader (10), and unsurprisingly this is an essential process; *trans*-splicing serving either to remove deleterious upstream sequences from the 5′ UTRs of mRNAs, or providing a cap to otherwise uncapped mRNAs generated from polycistronic RNAs (11,12). The two functions of spliced leader *trans*-splicing in *C. elegans* are partitioned into two mechanistically distinct families of spliced leader RNAs termed SL1 and SL2 RNAs. 5′ UTR removal is the main function of SL1 RNAs, while SL2 RNAs act in the resolution of operon transcripts, through a specific interaction between the SL2 RNA and the polyadenylation machinery (13–16).

We have previously identified proteins involved in SL1 *trans*-splicing using a *gfp*-reporter gene assay that detects inhibition of SL1 *trans*-splicing, and confirmed their involvement using reverse-transcription-qPCR (9). This has identified roles for Sm proteins, SNA-1, SNA-2 and to a lesser degree SUT-1, in SL1 *trans*-splicing.

The goal of the work presented here was to systematically investigate the molecular composition of the *C. elegans* SL1 snRNP and thereby better understand the mechanism by which SL1 sRNP interacts with both the pre-mRNA target and the spliceosome. We immunoprecipitated mature SL1 snRNP from embryonic extracts containing GFP-tagged endogenous SNA-1 and identified the RNA and protein components by RNA-Seq and label-free quantitative mass spectrometry, respectively. This confirmed that SNA-1 is an SL1 snRNP-specific protein and identified additional proteins associated with the SL1 snRNP. Using our GFP- and reverse-transcription-qPCR-based assays to monitor SL1 *trans*-splicing (9), we determined which of the interacting factors are required for spliced leader *trans*-splicing.

This led to the identification of SNA-3, a novel, essential, nematode-specific protein required for SL1 *trans*-splicing. SNA-3 has three NADAR domains and we show that at least two of these are required for function. We found that SNA-3 is connected to the SL1 snRNP by a combination of protein-protein and protein-RNA interactions, including factors involved in RNA metabolism such as the CBC-ARS2 complex components NCBP-1 and E01A2.2 (SRRT orthologue). Our findings indicate that SNA-3 may play a critical role in coordinating spliced leader *trans*-splicing with pre-mRNA transcription and the subsequent degradation of the Y-branch products of the *trans*-splicing reaction.

## MATERIALS AND METHODS

### Nematode and yeast strains

*C. elegans* strains were grown using standard culture conditions at 20°C, unless otherwise stated. The following previously published or Caenorhabditis Genetics Center sourced strains were used: N2 (wild type); PE793, *feIs11 [vit-2p::outron::gfp(M>A) myo-3p::mCherry]* X (17) and VC3472, *sna-3(gk3389)* III/*hT2[bli-4(e937) let-?(q782) qIs48]* (I;III).

The following strains were generated by CRISPR-mediated genome engineering, using the technique described in (17): PE906, *sna-1(fe75[GFP^3xFLAG::sna-1])* V; PE912, *sut-1(fe79[sut-1::GFP^3xFLAG])* II; PE918, *snr-2(fe82[GFP::3xFLAG::snr-2])* I; PE975, *uocIs1* II; *sna-3(fe92[sna-3::GFP^3xFLAG]) unc-119(ed3)* III; PE1015, *uocIs1 II; sna-3(fe110[sna-3::mNG^AID::3xFLAG])* *unc-119(ed3)* III. Two CRISPR engineered strains were created by SunyBiotech: PHX4149, *sna-3(syb4149)* III; PHX4538, *sna-3(syb4538) III/hT2[bli-4(e937) let-?(q782) qIs48] (I;III)*. Derivatives of PHX4149 were made to tag the mutant SNA-3 protein with mNeonGreen (mNG): PE1087, *sna-3(syb4149 fe129[sna-3::mNG^AID::3xFLAG])* III; PE1089, *sna-3(syb4149 fe131[sna-3::mNG^AID::3xFLAG])* III; PE1093, *sna-3(syb4149 fe135[sna-3::mNG^AID::3xFLAG])* III; and PE1094, *sna-3(syb4149 fe136[sna-3::mNG^AID::3xFLAG])* III. All four showed the same phenotype and expression pattern. Strains PE906, PE912 and PE918 were generated by injecting N2 hermaphrodites with a plasmid DNA cocktail consisting of 50 ng/μl pDD162 (source of Cas9), 50 ng/μl pRB1017-based guide RNA plasmid (18), 10 ng/μl pDD282-based homology repair template, 10 ng/μl pGH8, 5 ng/μl pCFJ104 and 2.5 ng/μL pCFJ90. PE975 and PE1015 were generated using the same approach, but we omitted pDD162 and injected HCL67, a transgenic strain that expresses Cas9 in the germline (19). PE1087, PE1089, PE1093 and PE1094 were generated using a revised CRISPR protocol (20). Details of guide RNAs and homology repair templates are given in Supplemental File 1.

Crosses were used to generate the following strains: PE1059, *sut-1(fe79[sut-1::GFP^3xFLAG])* II; *sna-3(gk3389)* III/*hT2[bli-4(e937) let-?(q782) qIs48]* (I;III); PE1060, *sna-3(gk3389)* III*/hT2[bli-4(e937) let-?(q782) qIs48]* (I;III); *sna-2(fe124[sna-2::GFP^3xFLAG])* IV; PE1061, *sna-3(gk3389)* III*/hT2[bli-4(e937) let-?(q782) qIs48]* (I;III); *sna-1(fe75[GFP^3xFLAG::sna-1])* V and PE1068, *sna-3(fe92[sna-3::GFP^3xFLAG]) unc-119(ed3)* III; *sna-2(tm2956) IV/nT1[qIs51]* (IV;V).

The *sna-3* loss-of-function rescuing assays were performed by injecting plasmid pESM3.15-1 (*eft-3p::sna-3*), along with the co-injection markers, pTG96 (*sur-5::gfp*; (21)), pRF4 (*rol-6(su1006)*; (22)) and/or *myo-2p::dTomato* (a generous gift from Rik Korswagen, Hubrecht Institute) into the VC3472 and PHX4538 strains. Injections and establishment of extrachromosomal lines was done as previously described (22,23) In both cases, rescuing lines were established, demonstrating that the *sna-3* mutations were responsible for the observed larval lethality. These strains were PE1010, *sna-3(gk3389) III; feEx337 [eft-3p::sna-3 myo-2p::dTomato rol-6(su1006)]*; PE1057*, sna-3(syb4538) III; feEx346[eft-3p::sna-3 sur-5::gfpmyo-2p::dTomato];* and PE1080, *sna-3(syb4149)* III*; feEx344[eft-3p::sna-3 sur-5::gfp myo-2p::dTomato]*.

Yeast strains Y187 (MATα, ura3-52, his3-200, ade2-101, trp1-901, leu2-3, 112, gal4Δ, met–, gal80Δ, MEL1, URA3::GAL1_UAS_-GAL1_TATA_-lacZ) and Y2HGold (MATa, trp1-901, leu2-3, 112, ura3-52, his3-200, gal4Δ, gal80Δ, LYS2::GAL1_UAS-_Gal1_TATA_–His3, GAL2_UAS_-Gal2_TATA_-Ade2, URA3::MEL1_UAS_–Mel1_TATA_ AUR1-C MEL1) were used for yeast two-hybrid interaction tests (Takara Bio Inc).

### Fluorescent microscopy

Worms were mounted in 5 μl M9 supplemented with 10 mM sodium azide on 5% agar pads. Images were obtained using a Zeiss Axioplan 2, equipped with a Hamamatsu Orca ER camera. Images were captured using Openlab 5 software. Post-processing was carried out using Fiji (24).

### Preparation of *C. elegans* embryo extracts

*C. elegans* embryo extracts were prepared essentially as described (25). Briefly, synchronised populations were prepared by alkaline hypochlorite treatment of gravid adult animals grown on NGM agar plates in the presence of OP50 bacteria, or in liquid culture in S-complete medium supplemented with OP50. Approximately 1.5 × 10^6^ embryos were transferred into 500 ml S-complete medium (50 mM potassium phosphate buffer (pH 6), 100 mM NaCl, 10 mM potassium citrate (pH 6), 3 mM CaCl_2_, 3 mM MgSO_4_ and 1× standard trace metal solution) supplemented with Nystatin (14 mg/ml) and grown overnight at 20°C in a shaking incubator at 160 rpm to L1 stage. After approximately 24 h, 14-16 g frozen OP50 bacteria were added and the incubation was continued until synchronous gravid adult worms emerged. Adults were recovered by centrifugation at 400 × g for 2 min, washed using MilliQ water, floated on 1 M sucrose and then washed again using MilliQ water to remove debris.

Alternatively, embryos were grown at 25°C on 10–12 14.5 cm NGM plates until they became gravid adults, essentially as described previously (26), except that plates were prepared with 2% agarose and seeded with live NA22 bacteria supplemented with the contents of a whole chicken egg. Animals were then recovered by adding MilliQ water and gentle agitation for 30 min followed by centrifugation and floating on sucrose as described above.

Animals were then lysed by alkaline hypochlorite treatment and embryos recovered by floating on sucrose. The embryos were washed three times with MilliQ water and twice in homogenisation buffer (10 mM Tris–HCl (pH 8.0), 1.5 mM MgCl_2_, 10 mM KCl, 1 mM DTT, 50 mM sucrose, 0.05% NP-40, 1X cOmplete EDTA-free protease inhibitor (Roche 1873560) and 1mM PMSF) and finally resuspended in 1 volume homogenisation buffer. Embryos were lysed using a Dura-Grind Dounce Tissue Grinder (Wheaton) and the extract was then cleared by centrifugation at 10 000 × g for 10 min at 4°C. The supernatants were dialysed against 20 mM Tris–HCl (pH 8.0), 50 mM KCl, 1 mM DTT, 10% glycerol, 0.5 mM EDTA, 1 mM PMSF at 4°C, and then flash-frozen in liquid nitrogen for storage at –80°C in an ultra-low temperature freezer.

### Immunoprecipitation of proteins

Extracts were supplemented with 35 mM KCl, 4 mM MgCl_2_, 1.5 mM DTT, 3% PEG 8000 (25). Where indicated, extracts were pre-treated with 200 units/ml RNase T1 and 5 units/ml RNase A (Invitrogen AM2286) for 1 h at 4°C.

Immunoprecipitations of GFP-tagged proteins were done in triplicate, using anti-GFP nanobody coupled agarose beads and control agarose beads (GFP-Trap and control agarose beads, Chromotek GmbH). Anti-GFP nanobody and control agarose beads were washed three times in wash buffer (10 mM Tris–HCl (pH 8), 100 mM KCl, 10 mM MgCl_2_, 5% glycerol, 0.25 mM EDTA, 3.5 mM DTT, 7.5% PEG6000, 0.5 mM PMSF). Normally, for each immunoprecipitation, 5 μl of settled agarose beads were mixed with 250 μl of extract treated with/without RNAses and incubated at 4°C. After 1 h, the beads were collected by centrifugation at 1500–2500 × g for 3 min at 4°C and washed twice with 500 μl of 20 mM Tris–HCl (pH8.0), 100 mM KCl, 0.1 mM EDTA, 0.1% NP-40, 1 mM DTT. Then they were transferred into fresh 1.5 ml tubes and washed three more times with the same buffer. After removal of the final wash, the wet beads were flash frozen in liquid nitrogen and stored at –80°C.

### Protein analysis by LC–MS/MS

Proteomic analysis was carried out as previously described (27). Briefly, proteins bound to beads were resuspended in 100 μl 50 mM ammonium bicarbonate, reduced in 2 mM dithiothreitol for 25 min at 60°C, S-alkylated in 4 mM iodoacetamide for 30 min at 25°C in the dark, then digested with 0.2 μg porcine trypsin (Promega sequencing grade) overnight at 37°C. The reaction was stopped by freezing at –70°C and drying by vacuum centrifugation. Peptides were dissolved in 40 μl 0.1% TFA, desalted using μ-C18 ZipTips (Merck Millipore) and eluted in 70% acetonitrile, 0.1% TFA. After drying by vacuum centrifugation, the peptides were dissolved in 10 μl 0.1% TFA for injection into a Q Exactive Plus nano LC–MS system and separation on a 75 μm i.d. × 25 cm PepMap RSLC C18 2 μm EASY-Spray column (Thermo Scientific). MS/MS data were acquired using a Top 10 data-dependent method (28).

### Quantitative protein analysis

Raw data files were processed using Maxquant version 1.6.5.0 (29), with default settings unless stated otherwise. The inbuilt Andromeda search engine (30) was used for protein identification against the *C. elegans* reference proteome UP000001940_6239 (downloaded 11/04/2019). Carbamidomethylation of cysteine was used as fixed modification; oxidation of methionine and acetylation of the protein N terminus were set as variable modifications. Instrument selection was Orbitrap and Trypsin/P cleavage specificity was used. The minimal peptide length was seven amino acids and a maximum of two missed cleavages were allowed. FTMS MS/MS match tolerance was set to 10 ppm and ITMS MS/MS match tolerance to 0.06 Da. False discovery rate was 1% for peptide and protein identifications. Each protein group was required to contain at least two unique or razor peptides, with each peptide used only once for protein identification (Razor protein FDR). For label-free quantification (LFQ) (31) the LFQ minimum ratio count was set to two and both unique and razor peptides were used for quantification. The match from and to option was used as Match type.

Perseus software (version 1.6.5.0) (32) was used for data analysis. Proteins missing in at least one of three immunoprecipitations with anti-GFP nanobody coupled agarose beads were excluded. The LFQ intensities were log_2_-transformed and missing values were replaced with low abundance values based on simulated normal distribution using Perseus standard settings. Then, the differences between protein amounts in immunoprecipitations using anti-GFP nanobody beads versus control precipitations using agarose beads were examined using a two-sample *t*-test with a permutation-based FDR, and visualised using volcano plots with a conservative q-value *<*0.01 and an S0 value of 2.5 to highlight clearly enriched proteins. Volcano plots were drawn using GraphPad Prism version 5 for Windows, GraphPad Software, San Diego, California USA, www.graphpad.com.

### RNA immunoprecipitation sequencing (RIP-Seq)

Immunoprecipitations were repeated in triplicate using anti-GFP nanobody coupled agarose beads or control agarose beads as described, except that 750 μl extract and 20 μl settled agarose beads were used. The wet pellets were treated with 200 μl 0.2 mg/ml Proteinase K in 200 mM Tris–HCl (pH7.5), 300 mM NaCl, 2% SDS and 25 mM EDTA at 65°C for 15 min. Then the RNA was recovered by two subsequent extractions with acidic phenol:chloroform:isoamylalcohol (125:24:1, pH 4.5), supplemented with 10 μg linear acrylamide and concentrated by ethanol precipitation, washed with 80% ethanol and air dried.

Library preparation, sequencing and analysis were done at the Centre for Genome Enabled Biology and Medicine (CGEBM; University of Aberdeen). Libraries were prepared using the Diagenode CATS small RNA sequencing kit, following the manufacturer's instructions, and sequenced on an Illumina NextSeq500 high-output v2.5 flow cell producing 1 × 86 bp reads. Illumina adapters, as well as technical sequences detailed in the CATS small-RNA manual (polyA/T tails; template-switching motif; CATS adapters), were removed from the data using CUTADAPT 1.16 (33). Poor-quality bases with a phred score below 20 were trimmed and reads shorter than 18 bp after trimming were discarded. The quality-filtered reads were aligned to the *C. elegans* genome (ENSEMBL WBcel235 assembly) using HISAT 2.1.0 (34) and quantified against gene annotations using FEATURECOUNTS 1.6.1 (35). Multi-mapping reads were assigned to all mapping locations and each location received a fractional count. Gene-based read counts were analysed in R using the DESEQ2 package (36) and variance stabilized normalized counts were established. Expression fold-changes were identified with negative binomial models, shrinking fold changes of low-count genes with the default method (36) and correcting *P*-values for multiple testing using the false-discovery rate (FDR) method (37). Genes of interest (snoRNAs, snRNAs and ncRNAs) were downloaded using the WormMine tool.

### RNA interference and monitoring of SL1 *trans*-splicing using a *gfp* reporter gene

To prepare the RNAi feeding vectors, DNA was PCR amplified from genomic DNA or cDNA and inserted by In-Fusion cloning (Takara Bio Inc) into the plasmid pPD129.36 cleaved using appropriate restriction enzymes. Primer sequences and restriction enzymes used are listed in Supplemental File 1. Primers were purchased from Sigma-Aldrich (now Merck). RNA interference by feeding was done in PE793 animals with a *gfp* reporter gene that is activated when SL1 *trans*-splicing is impaired, and the proportion of GFP fluorescent animals was determined as described previously (9,38).

### Monitoring SL1 *trans*-splicing using qPCR

SL1 *trans*-splicing of the *gfp* reporter and of *rps-3* mRNA was analysed by reverse transcription of total RNA isolated from PE793 exposed to RNAi by feeding as described previously (9). *unc-22(RNAi)* known not to inhibit SL1 *trans*-splicing was used as a negative control, *sna-1(RNAi)* or *sna-2(RNAi)* (treatments known to inhibit SL1 *trans*-splicing) were used as positive controls. Between 4000 and 6000 animals were exposed to RNAi treatments, harvested, washed with M9 buffer, mixed with TRIzol and snap-frozen in liquid nitrogen. Total RNA was then isolated using the PureLink RNA Mini kit (Life Technologies) with modifications for TRIzol treated samples and DNAse treatment as described by the manufacturer. Normally, 1 μg total RNA was reverse transcribed to cDNA using oligo(dT) Primers (Promega) and Superscript II or Superscript III Reverse Transcriptase (Invitrogen) in a volume of 20 μl, according to the manufacturer's instructions. The qPCR assays, including primer sequences and Universal ProbeLibrary probes (Roche) used to monitor SL1 *trans*-splicing of *gfp* reporter gene and *rps-3* transcripts, were performed as described previously (9). Briefly, the assays are designed to measure the levels of non-*trans*-spliced *gfp* or *rps-3* transcripts, standardised using qPCR assays that measure total *gfp* or *rps-3* transcript levels, respectively.

qPCR reactions were set up manually, using the LightCycler 480 Probes Master mix (Roche) according to manufacturer's instructions, and analysed using a Roche LightCycler 480, with the standard programme for “Mono Color Hydrolysis Probe/UPL”. The qPCR reactions were analysed using LightCycler 480 software release 1.5.1.62 Sp3 or earlier. Inhibition of SL1 *trans*-splicing was detected by comparison to the control *unc-22(RNAi)* treatment using the comparative CT method (39). qPCR reactions were done as technical triplicates.

### Yeast two-hybrid assays

Open reading frames were amplified from cDNA prepared by reverse transcription of *C. elegans* total RNA using CloneAmp DNA polymerase (Takara Bio Inc) and the primers used are listed in Supplemental File 1. Amplicons were inserted into either pGBKT7 or pGADT7 cleaved with EcoRI and BamHI by In-Fusion cloning (Takara Bio Inc). The sequences were confirmed by Sanger sequencing (DNA sequencing and Services, Dundee University and Eurofins Genomics). pGADT7 and derivatives were transformed into yeast strain Y187 and pGBKT7 and derivatives into yeast strain Y2HGold (Takara Bio Inc.). Yeast transformation and subsequent mating of transformants were done using standard protocols. Protein-protein interactions were assayed by comparing growth on synthetic defined medium lacking leucine and tryptophan with growth on synthetic defined medium lacking in addition adenine and histidine.

### Western blotting

Animals were either hand-picked, or rinsed from plates and washed in M9 buffer, and flash frozen. Prior to SDS-PAGE, thawed animals were resuspended in SDS-PAGE loading buffer, subjected to 3 min sonication in an ultrasonic bath (Ultrawave, 100 W) and heated to 95°C for 5 min. SDS-PAGE was performed using NuPAGE 4–12% gradient gels with MOPS SDS as buffer system (Invitrogen). Proteins were transferred onto Hybond-P membranes (Cytiva) by electro blotting and membranes were probed as indicated with either mouse anti-GFP antibodies (Roche 11814460001), mouse anti-mNeonGreen antibodies (Chromotek) or mouse anti-GAPDH antibodies (Invitrogen AM4300). Proteins were detected using HRP-linked anti-mouse IgG antibodies (Cell Signalling Technology) with Immobilon Forte Western HRP substrate (Merck) and visualised using an iBright FL1000 imaging system (Invitrogen). Membranes were also stained with amido black (Merck). Pre-stained broad range molecular weight marker was from New England Biolabs. Quantitation was done using Fiji (24).

### Graphical representation of protein structures

Graphics of protein structures were done using The PyMOL Molecular Graphics System, Version 2.0 Schrödinger, LLC.

## RESULTS

### RIP-Seq analysis demonstrates that SNA-1 is an SL1 snRNP-specific protein

To investigate the molecular composition of the SL1 snRNP we modified the chromosomal copy of the *sna-1* gene so that it expresses an N-terminal GFP tagged version of the SNA-1 protein (17). The resultant strain, PE906, displays GFP fluorescence in all somatic and germline nuclei, with expression being strongest in actively dividing cell lineages (see Supplemental Figure S5). This expression pattern is consistent with the previous work showing that SNA-1 is a key component of the nematode SL1 *trans*-splicing machinery (7,9).

Since SNA-1 is a proposed SL1 snRNP component, our first goal was to confirm that it is associated with the SL1 RNA. To do this, we identified the RNAs associated with SNA-1 by immunoprecipitation followed by RNA-Seq (RIP-Seq). We prepared extracts from PE906 embryos based on a protocol established by the Blumenthal laboratory (25). The SL1 snRNP was immunoprecipitated using anti-GFP-nanobodies coupled to agarose beads, and immunoprecipitations were compared to control precipitations performed with agarose beads only.

RIP-Seq analysis shows that SL1 RNAs (products of specific *sls-1* loci) were enriched between 4.13- and 34.29-fold in SNA-1 immunoprecipitations (Table 1 and Supplemental File 2). Other RNAs enriched up to four fold include a few mRNAs, the snoRNA ZK185.9 (3.61-fold enriched) and SmY-8 RNA (1.8-fold enriched), a member of the SmY family of non-coding RNAs that have previously been implicated in spliced leader *trans*-splicing (3,8). Importantly, neither SL2 RNAs nor spliceosomal snRNAs such as U1 or U2 snRNA, were significantly enriched (Supplemental File 2). Taken together, these data are consistent with that SNA-1 is an SL1 snRNP-specific protein.

**Table 1.** SNA-1 RIP-Seq enriches SL1 RNA. Immunoprecipitations using embryonic extract from PE906 animals expressing GFP-tagged SNA-1 were done using anti-GFP nanobodies coupled to agarose beads or control agarose beads, and performed in triplicates. RNA was isolated from immunoprecipitates, sequenced and analyzed as described in Materials and Methods. The table shows mean normalized read counts and linear fold changes of RNAs significantly enriched in SNA-1 immunoprecipitations (*q*-value ≤ 0.05)

Transcript	Mean normalized counts	Linear fold change (anti-GFP/control)	*P*-value	*q*-value
*sls-1.12*	41275.19	34.30	2.2E-108	2.4E-104
*sls-1.7*	5230.04	21.04	1.8E-72	9.9E-69
*sls-1.6*	5237.53	19.63	1.4E-69	5.1E-66
*sls-1.10*	7025.28	10.71	4.0E-45	1.1E-41
*sls-1.11*	7299.14	10.37	3.4E-44	7.3E-41
*sls-1.8*	3044.66	4.20	8.2E-27	1.5E-23
*sls-1.3*	3023.15	4.15	3.3E-26	4.0E-23
*sls-1.9*	3035.79	4.15	1.9E-26	2.6E-23
*sls-1.2*	3041.35	4.14	1.6E-26	2.5E-23
*sls-1.4*	3018.90	4.13	5.8E-26	6.3E-23
*ZK185.9*	1440.99	3.61	9.6E-21	9.5E-18
*R04F11.2*	316.72	2.88	1.0E-19	9.4E-17
*vit-2*	42.94	2.46	2.4E-06	1.6E-03
*zig-1*	81.94	2.43	7.2E-10	6.0E-07
*tbx-9*	63.92	2.22	4.6E-08	3.6E-05
*clp-4*	62.40	2.15	2.2E-06	1.6E-03
*T03F1.6*	51.92	2.00	3.2E-05	1.8E-02
*smy-8*	88.42	2.00	3.2E-06	2.0E-03
*sup-36*	155.57	1.80	1.5E-05	9.1E-03
*dao-2*	451.96	1.53	6.6E-05	3.6E-02
*htz-1*	777.25	1.43	9.4E-05	4.9E-02

### GFP::SNA-1 immunoprecipitation identifies core SL1 snRNP components

**Figure 1.**
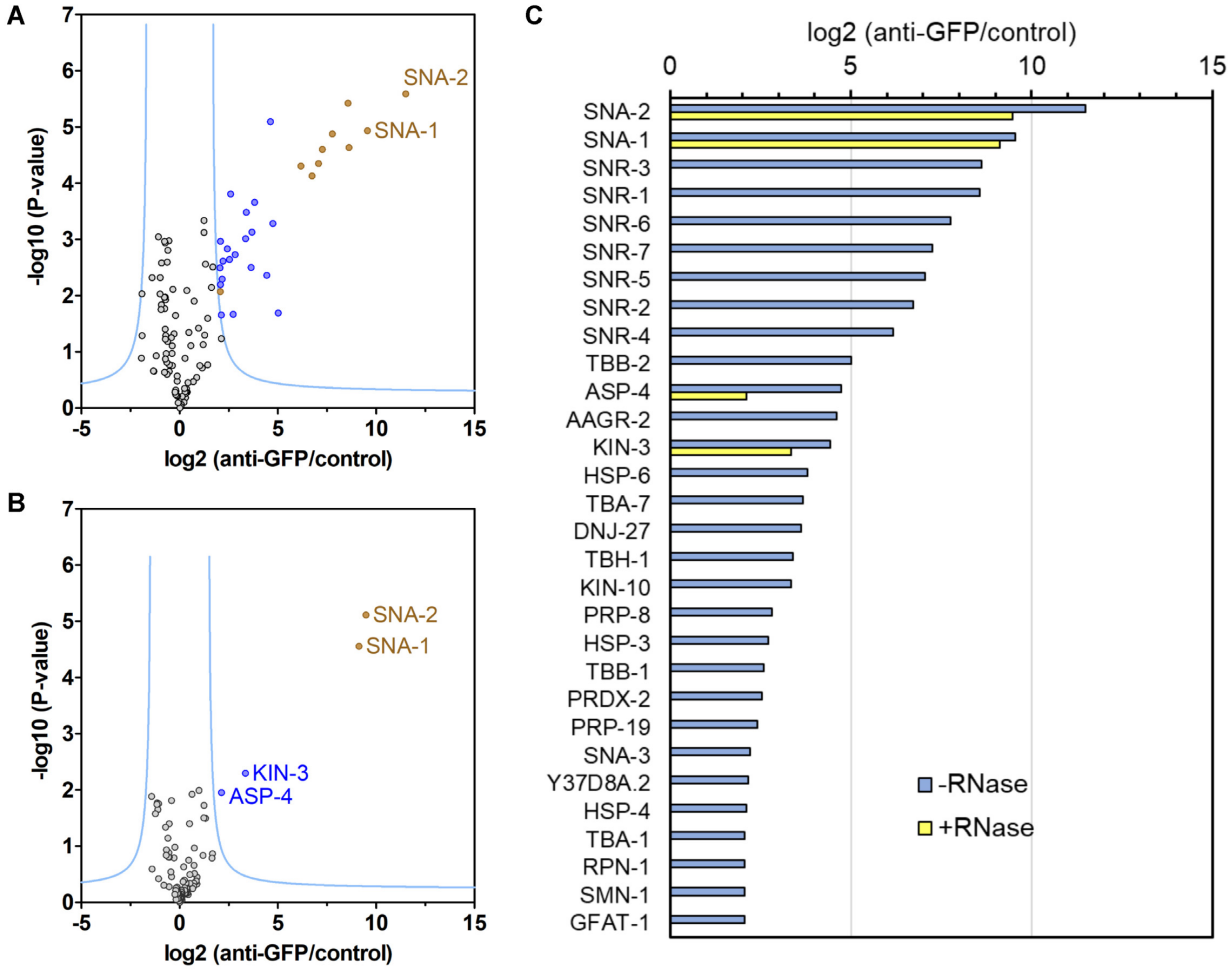
Protein composition of the SL1 snRNP: RNA-dependent and independent interactions with SNA-1. (**A**) Volcano plot showing proteins enriched by co-immunoprecipitation with SNA-1. Immunoprecipitations were done using embryonic extract from PE906 animals expressing GFP-tagged SNA-1 and anti-GFP nanobodies coupled to agarose beads, or using control agarose beads. Immunoprecipitations were done in parallel in triplicates, and the immunoprecipitated proteins were analysed by label-free quantification using MaxQuant and Perseus software as described. Proteins were plotted showing the enrichment in GFP::SNA-1 immunoprecipitations compared to controls (log2 (anti-GFP/control)), and the false discovery rate (–log_10_ (*P*-value)). The significance cutoff curve is drawn in blue (FDR **<** 0.01 and S0 of 2.5). Significantly enriched proteins shown in gold are SNA-1 and SNA-2 proteins, the seven SNR proteins forming the Sm protein ring and SMN-1 linked to SL1 *trans*-splicing (9). Proteins shown in blue have not been previously implicated in spliced leader *trans*-splicing. (**B**) Volcano plot showing proteins interacting with SNA-1 in an RNA-independent manner. Immunoprecipitations were done and analysed as described under (A) except that the extract was pre-treated with RNase A/T1 prior to immunoprecipitation. (**C**) Summary of proteins significantly enriched in immunoprecipitations from extracts treated without/with RNAses shown in (A) and (B). The fold enrichment in SNA-1 immunoprecipitations is plotted on the X-axis (log_2_ scale, anti-GFP/control). Proteins enriched in immunoprecipitation from extract treated without/with RNAse A/T1 are shown in blue and yellow, respectively. The proteins are identified by abbreviations based on their gene names, or if the gene has not been named, the open reading frame identifier. Note that SNA-3 and PRDX-2 only appear with their Uniprot identifiers Q9GYR5 and A0A0K3AUJ9, respectively, in Supplemental File 3 summarising the results of the analyses done using PERSEUS.

To determine the SL1 snRNP protein composition, the proteins that co-immunoprecipitated with SNA-1 in embryonic extract were identified and quantified. Proteins were released from immunoprecipitates and control beads by digestion with trypsin, and then analysed by LC–MS/MS. Label free quantitation of proteins was then performed using MaxQuant software and the results were analysed using the Perseus tool (29–32). The volcano plot in Figure 1A shows the 112 proteins that were identified using this approach, with 25 proteins significantly enriched (see Supplemental File 3 for details). To confirm that any enrichment is specifically due to the immunoprecipitation of GFP-tagged SNA-1, we performed a control experiment using embryonic extract prepared from wild type N2 animals (Supplemental Figure S1, File 3). We did not detect a significant enrichment of any proteins, confirming that any enrichment is specifically due to the immunoprecipitation of GFP-tagged SNA-1.

Significantly enriched proteins in SNA-1 immunoprecipitations (Figure 1A) are, as expected, SNA-1 protein itself; the previously identified SL1 snRNP component, SNA-2; the seven SNR proteins that form the Sm protein ring bound to SL1 RNA; and SMN-1, the orthologue of SMN involved in Sm protein assembly, nuclear import and subnuclear localisation of snRNPs and the assembly of the spliceosome (8,9,40,41). SMN-1 has previously been shown to be involved in spliced leader *trans*-splicing (9).

### Other proteins associated with the SL1 snRNP

Proteins shown in blue in Figure 1A may be related to SL1 *trans*-splicing and are named in the summary graph in Figure 1C (the full analysis of immunoprecipitations is shown in Supplemental File 3). These include splicing factors (PRP-8, PRP-19), tubulins (TBA-1/TBA-4, TBA-7, TBB-1, TBB-2), molecules involved in protein folding and degradation (DNJ-27, HSP-3, HSP-4, HSP-6, ASP-4, RPN-1), metabolic enzymes (AAGR-2, GFAT-1, TBH-1 and Y37D8A.2, an ortholog of human PLBD2), orthologs of the catalytic and regulatory subunits of casein kinase II CSNK2A1 and CSNK2B (KIN-3, KIN-10), an ortholog of human peroxiredoxin 1 (PRDX-2) and a novel, nematode-specific protein (Uniprot identifier Q9GYR5), which is encoded by the gene C23G10.8. This latter protein, we named SNA-3 (small nuclear RNA associated), based on its association with SL1 RNA and on the fact that its loss of function results in defects in SL1 *trans*-splicing (see below).

### The interaction between SNA-1 and SNA-2 is resistant to treatment with RNAse

Since we were investigating a ribonucleoprotein, proteins that interact with SNA-1 could be dependent upon protein-protein and or protein–RNA interactions. To determine whether the interaction of these proteins with SNA-1 is dependent on RNA, embryonic extract was treated with a mixture of RNase A and RNase T1 prior to the immunoprecipitation step (Supplemental Figure S2). As shown in Figure 1B and C, this greatly reduced the number of enriched factors: only SNA-2, KIN-3 and ASP-4 were significantly enriched together with SNA-1, indicating that the interaction of SNA-1 with SNA-2 and these two other proteins is RNA independent. This result and the yeast two-hybrid data shown below, confirm the direct interaction between SNA-1 and SNA-2 described previously (7,8).

### Loss of PRP-19 and SNA-3 impairs SL1 *trans*-splicing

**Figure 2.**
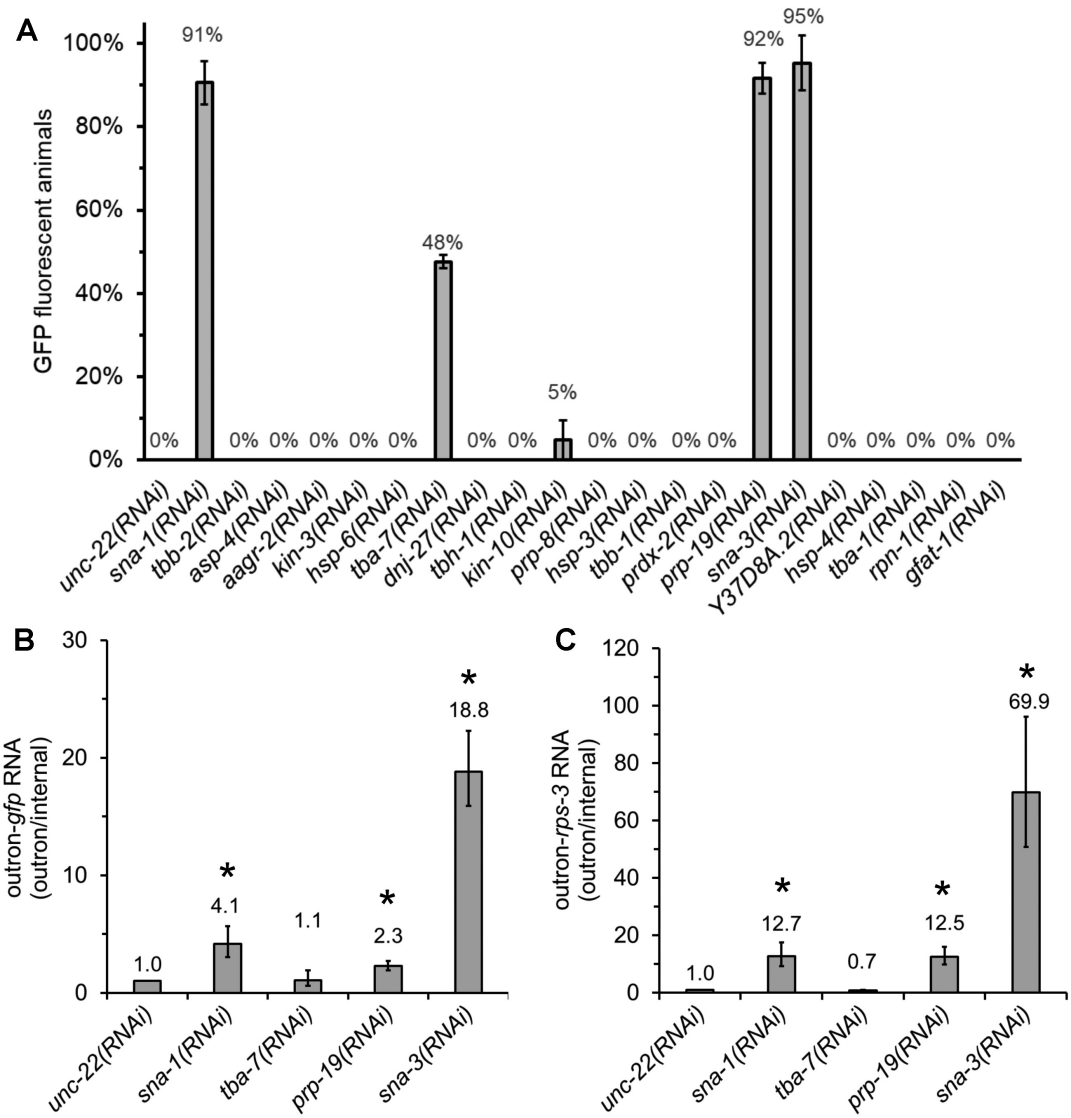
Identification of new SL1 snRNP-associated factors required for SL1 *trans*-splicing by RNAi knockdown. (**A**) Quantitation of GFP expression in PE793 animals carrying the P*vit-2*::outron::GFP^M1A^ transgene subjected to RNAi treatments; activation of the *gfp*-reporter gene indicates inhibition of SL1 *trans*-splicing. The percentage of GFP fluorescent animals was determined by fluorescence microscopy. Error bars represent the standard deviation based on three independent experiments. Percentages were rounded to the nearest whole percentage. (B, C) Detection of SL1 t*rans*-splicing inhibition by RT-qPCR; elevated outron-*gfp* or outron-*rps-3* levels indicate inhibition of SL1 *trans*-splicing. Animals carrying the P*vit-2*::outron::GFP^M1A^ transgene were subjected to RNAi and *trans*-splicing of the *gfp* reporter gene (**B**) and *rps-3* transcripts was analyzed by reverse transcription followed by qPCR. Outron-*gfp* and outron-*rps-3* RNA levels were standardized with respect to an internal part of the mRNA (internal), with levels in *unc-22(RNAi)* animals defined as 1. Error bars show standard deviation from three technical replicates. * indicates treatments where SL1 *trans*-splicing is significantly inhibited compared to *unc-22(RNAi)* (*t*-test, *P* ≤ 0.05). Similar results were obtained in two independent experiments.

To determine the functional significance of the interactions that we have discovered, and to establish whether the new proteins identified are required for SL1 *trans*-splicing, we examined the effect of depleting them through RNAi. We used a *C. elegans* transgenic strain that expresses GFP in response to inhibition of spliced leader *trans*-splicing (9). RNAi knockdown of the Sm proteins, SNR-1 to SNR-7; as well as SMN-1, SNA-1, SNA-2 and SUT-1 have previously been shown by us to affect SL1 *trans*-splicing, so we repeated the analysis here only with *sna-1(RNAi)* as a positive control. Of the proteins identified from the immunoprecipitations, RNAi knockdown of SNA-3, PRP-19 and TBA-7 led to a robust GFP signal from the *trans*-splicing reporter, with SNA-3 and PRP-19 loss-of-function giving similar levels of expression to that observed in positive control, *sna-1(RNAi)* animals (Figure 2A). RNAi knock-down of all other GFP::SNA-1 interacting proteins gave fluorescence signals indistinguishable from the negative control, *unc-22(RNAi)*.

To confirm that the *gfp* reporter gene activation was caused by an inhibition of SL1 *trans*-splicing, we analysed SL1 *trans*-splicing of mRNA derived from the *gfp* reporter gene and from the endogenous *rps-3* gene by reverse transcription followed by qPCR (9). Our assay measures the level of non-*trans*-spliced (outron retaining) mRNA, relative to the level of total mRNA, and results are standardised with respect to *trans*-splicing detected in *unc-22(RNAi)* treated animals (9). We again used *sna-1(RNAi)* as a positive control (9). We assayed *sna-3*, *prp-19* and *tba-7*, since their knock-down caused clear GFP expression (Figure 2A), but excluded *kin-10* as *gfp* activation in response to RNAi knock-down of this gene was marginal. We observed a strong inhibition of SL1 *trans*-splicing for both *gfp* and *rps-3* transcripts in response to *sna-3* or *prp-19* RNAi, while depletion of *tba-7* transcripts had no detectable effect (Figure 2B, C). In conclusion, this analysis demonstrates a critical role for the splicing factor PRP-19 in SL1 *trans*-splicing and identifies SNA-3 as a novel factor involved in this process.

### SNA-3 is a novel essential factor involved in SL1 *trans*-splicing

**Figure 3.**
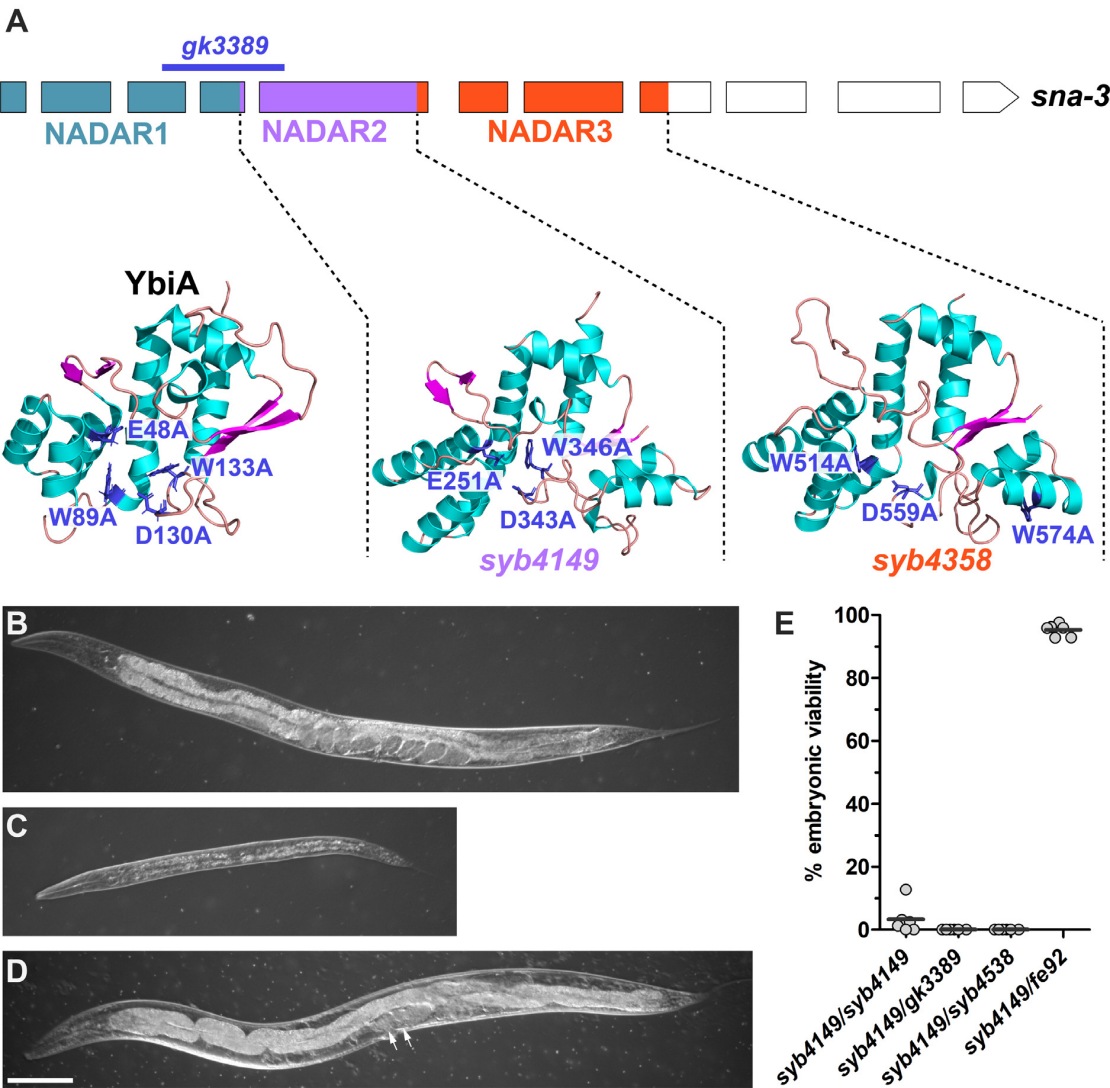
SNA-3 is an essential NADAR domain containing protein. (**A**) Schematic of the *sna-3* gene structure, showing the regions encoding the three NADAR domains (coloured shading). The region deleted by the *gk3389* loss-of-function mutation is indicated by the line. Boxes represent protein coding exons (the 3′ UTR is omitted). The structure of the *E. coli* YbiA protein (PDB ID 2B3W; Ramelot *et al*, unpublished data) and the AlphaFold structure predictions for the two SNA-3 NADAR domains are shown below the gene (59). The key mutations that abolish N-glycosidase activity in YbiA and their homologues in the SNA-3 domains are indicated. Note that W574 does not have a homologue in YbiA, but the mutation was made prior to the availability of the AlphaFold prediction. (**B**) Wild-type phenotype displayed by a representative *sna-3(gk3389)/+* heterozygous adult hermaphrodite compared to (**C**), the developmentally arrested larval phenotype of a representative *sna-3(gk3389)* homozygote at the same post-hatching age. (**D**) *sna-3(syb4149)* homozygous adult raised at 25°C showing partially sterile phenotype: one gonad arm appears functional, the uterus containing two viable embryos (arrows). Scale bar represents 100 μm. (**E**) Fourth larval stage (L4) animals of each genotype were transferred to the restrictive temperature (25°C) and their offspring scored for embryonic viability. The broods of six animals for each genotype were scored. The horizontal bar indicates the mean for each dataset.

*sna-3* is an essential gene encoding a 937 amino acid protein with three predicted ‘NADAR’ (NAD and ADP-ribose) domains (Figure 3A) (42), which has yet to be linked to a specific cellular function. A predicted null allele (*gk3389*) confers recessive larval lethality, the affected animals showing multiple defects in postembryonic cell proliferation (Figure 3A, C). The larval lethality is fully rescued by an extrachromosomal transgene consisting of the *sna-3* gene under the control of the *eft-3* promoter. Rescued transgenic animals reach fertile adulthood.

SNA-3 is conserved throughout the nematodes and no homologues are detectable outside the phylum. The phylogeny of individual SNA-3 NADAR domains demonstrates clear domain-specific sequence conservation preserved across the major nematode groups for which we have genome sequence data (Supplemental Figure S3). The broad conservation within, and lack of detectable homologues beyond the Nematoda are features consistent with a protein specific to nematode spliced leader *trans*-splicing.

The function of the NADAR domain is not well defined; it has been conjectured, based on its association with known proteins or domains, to be involved in RNA metabolism events involving NAD or ADP-ribose derived molecules (42). However, the only definitive biochemical activity demonstrated for specific NADAR domain containing proteins including the *Escherichia coli* YbiA protein is as N-glycosidases deployed for the removal of intermediates in riboflavin biosynthesis (43). This study also identified four residues that are critical for YbiA N-glycosidase activity (Figure 3A). To determine the importance of the individual NADAR domains for SNA-3 function, we investigated the phenotypic consequences of mutating the homologues of these residues that we were able to identify in the second and third NADAR domains of SNA-3 (Figure 3A, NADAR2 and NADAR3). These residues are not conserved in NADAR domain 1 so we were not able to reliably test the role of this domain.

We determined the consequences of mutating the functionally conserved residues in NADAR domain 2 (E251A, D343A and W346A). *sna-3(syb4149)* homozygotes raised at permissive temperatures 16°C and 20°C show wild type fertility (mean brood sizes [*n* = 5] at 16°C and 20°C were 303 and 314, respectively) but when grown at 25°C are almost completely sterile (82.5%; *n* = 185) (Figure 3D). This sterility was rescued by transgenic copies of the *sna-3* gene expressed from the *eft-3* promoter.

In contrast, mutating the three residues (W514A, D559A and W574A) in NADAR domain 3, creating *sna-3(syb4538)*, conferred the same recessive larval lethality that we observed for the *sna-3* deletion allele. This larval lethality was similarly rescued by a transgenic copy of the *sna-3* gene under the control of the *eft-3* promoter.

To further investigate the hypomorphic nature of the *sna-3(syb4149)* allele, we made heteroallelic combinations of this mutation with the two putative null alleles, *gk3389* and *syb4538*. Heteroallelic L4 larvae transferred to 25°C produced broods that showed 100% embryonic lethality. This is similar to the phenotype seen in similarly treated *sna-3(syb4149)* homozygotes (Figure 3E), indicating that at the restrictive temperature the *syb4149* mutations strongly impair SNA-3 function.

We also took advantage of this phenotype to assess the function of the GFP-tagged SNA-3 protein (see below): *syb4149/fe92[sna-3::GFP^3xFLAG]* heteroallelic animals show wild type viability, indicating that the addition of the GFP-tag to the C-terminus of SNA-3 does not detectably alter its function.

### Yeast two-hybrid assays detect interactions between SNA-1 and SNA-2 and between SUT-1 and SNA-3

To confirm and extend our knowledge of the physical interactions between SNA-1, SNA-2 and SNA-3, we performed yeast two-hybrid assays. We also included SUT-1, the paralogue of SNA-1 (44), since this protein has previously been shown to interact with SNA-2 in immunoprecipitations from embryonic extracts (8).

**Figure 4.**
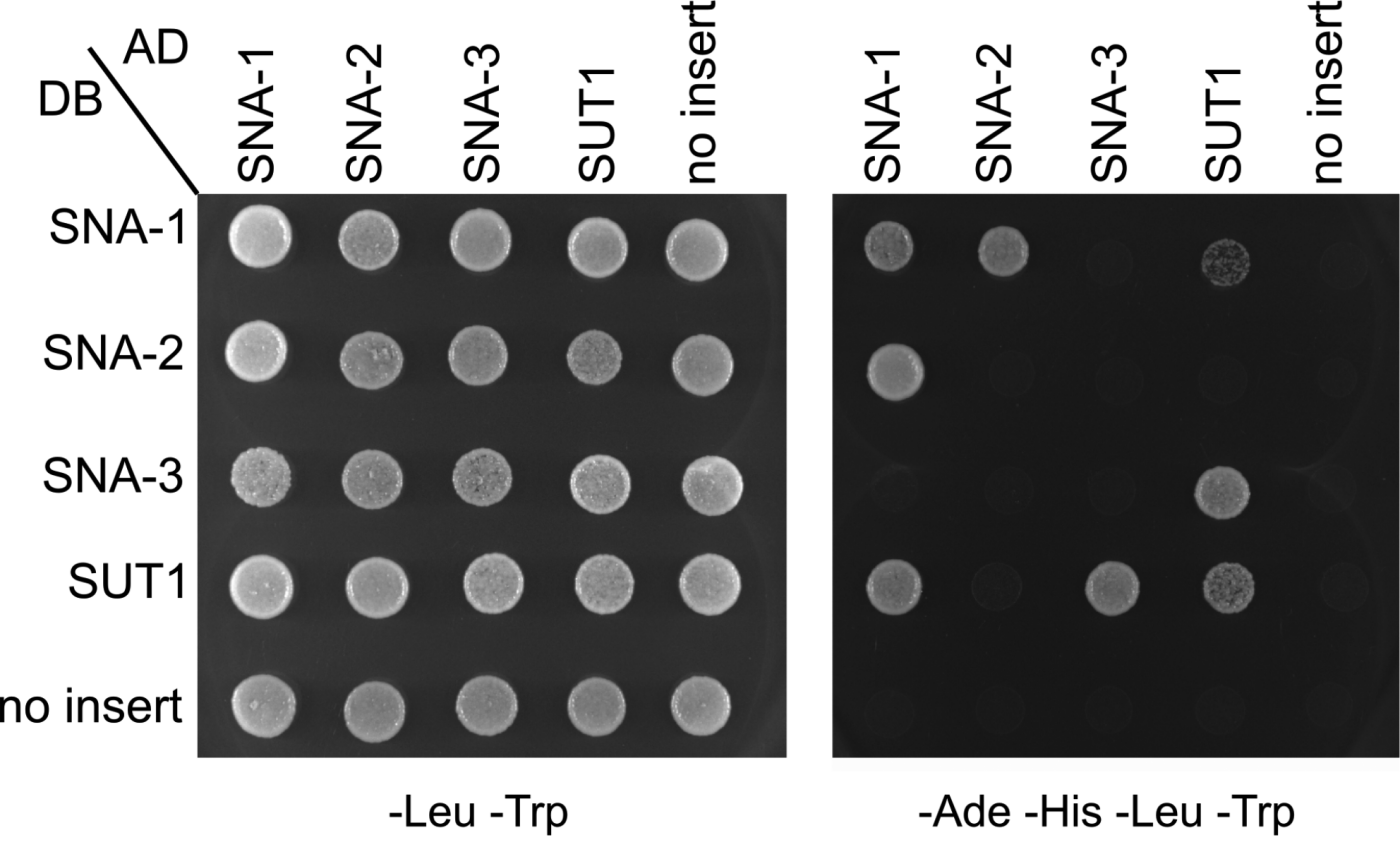
Protein-protein interactions between SNA-1, SNA-2, SNA-3 and SUT-1 revealed by yeast two-hybrid assays. Interactions were detected by expressing SNA-1, SNA-2, SUT-1 and SNA-3 as Gal4 DNA-binding domain fusion proteins (DB) using pGBKT7 derivatives in Y2HGold yeast, and as Gal4 activation domain fusion proteins (AD) from pGADT7 derivatives in Y187 yeast. No-insert controls were done with unmodified pGBKT7 or pGADT7. Diploids were plated as controls on synthetic defined yeast medium without leucine or tryptophan (-Leu -Trp) and on synthetic defined yeast medium without adenine, histidine, leucine and tryptophan (-Ade -His -Leu- Trp) to test for the activation of the Ade2 and His3 reporter genes that are under the control of Gal regulatory elements recognised by the DB fusion proteins.

We confirmed the direct interaction between SNA-1 and SNA-2 that was expected based on data from Figure 1 and found that SUT-1 can interact with SNA-3 (Figure 4). However, we were not able to detect direct interactions between SNA-2 and SUT-1, or between SNA-1 and SNA-3. We also found that, at least when expressed in yeast, SNA-1 and SUT-1 can self-interact and interact with one another, possibly a reflection of their shared ancestry (Figure 4).

SNA-1 and SUT-1 have undergone significant sub-functionalization, as judged by their primary sequences, but they have retained significant sequence similarity at their N-termini, with AlphaFold predictions indicating that this region forms an alpha-helical hairpin (Supplemental Figure S4) of unknown function. It was previously proposed that SNA-1 and SUT-1 interact with SNA-2 through this motif (8), but we found that deleting this motif had no effect on the interactions between SNA-1 and SNA-2, or between SUT-1 and SNA-3 (Supplemental Figure S4).

### Nuclear Localisation of SNA-1 and SUT-1 is dependent on SNA-2 and SNA-3, respectively

To examine the subcellular localisation of the novel *trans*-splicing factor SNA-3, and compare it to the localisation of SNA-1, SNA-2, and SUT-1; proteins known to be involved in SL1 *trans*-splicing but not yet examined in terms of sub-cellular localisation (8,9), we generated strains that express GFP-tagged proteins from their respective chromosomal genes. All four fusion proteins gave essentially identical expression patterns, being expressed in the nuclei of all somatic and germline cells, consistent with their proposed functions in spliced leader *trans*-splicing (Supplemental Figure S5).

**Figure 5.**
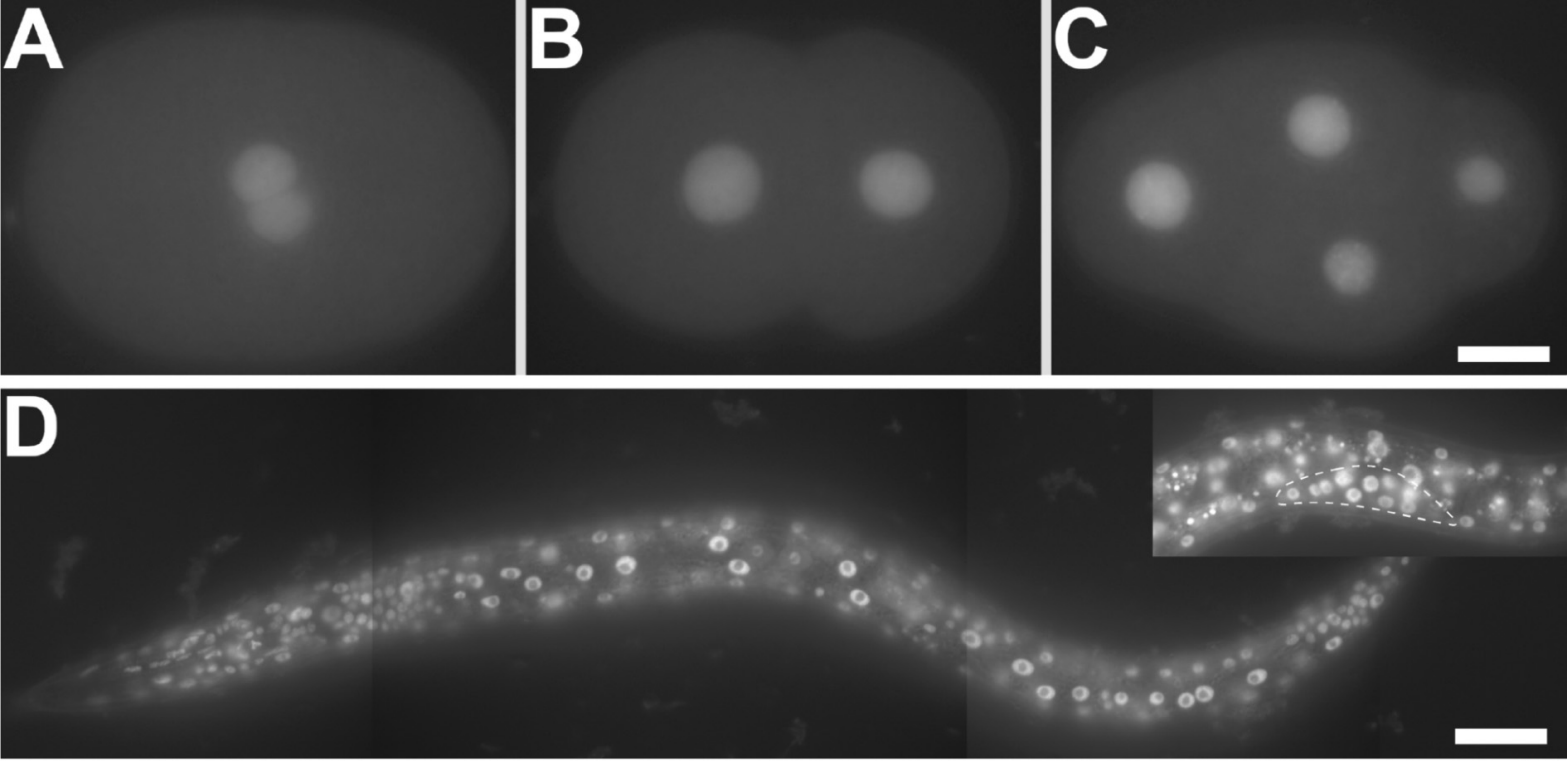
SNA-3::GFP is expressed in somatic and germline nuclei throughout development. (A–C) SNA-3::GFP in the sperm and oocyte nuclei of the one cell embryo (**A**), and in the zygotic nuclei of two and four cell embryos (**B** and **C**, respectively). (**D**) Somatic cells expressing SNA-3::GFP in the second larval stage (L2). Inset shows expression in germline nuclei (dotted outline). Scale bar represents 10 μm (A–C) or 20 μm (D).

We examined the expression pattern of SNA-3::GFP throughout development (Figure 5). The fusion protein is found in the paternal and maternal pronuclei in one-cell embryos (Figure 5A) and expression is maintained throughout early embryogenesis, even in cells that are transcriptionally quiescent, indicating substantial parental contributions of the protein to the embryo (Figure 5B, C). Somatic and germline cells express nuclear-localised SNA-3::GFP throughout postembryonic development, with expression being particularly strong in actively dividing cells (Figure 5D), presumably reflecting the high demand for spliced leader *trans*-splicing.

**Figure 6.**
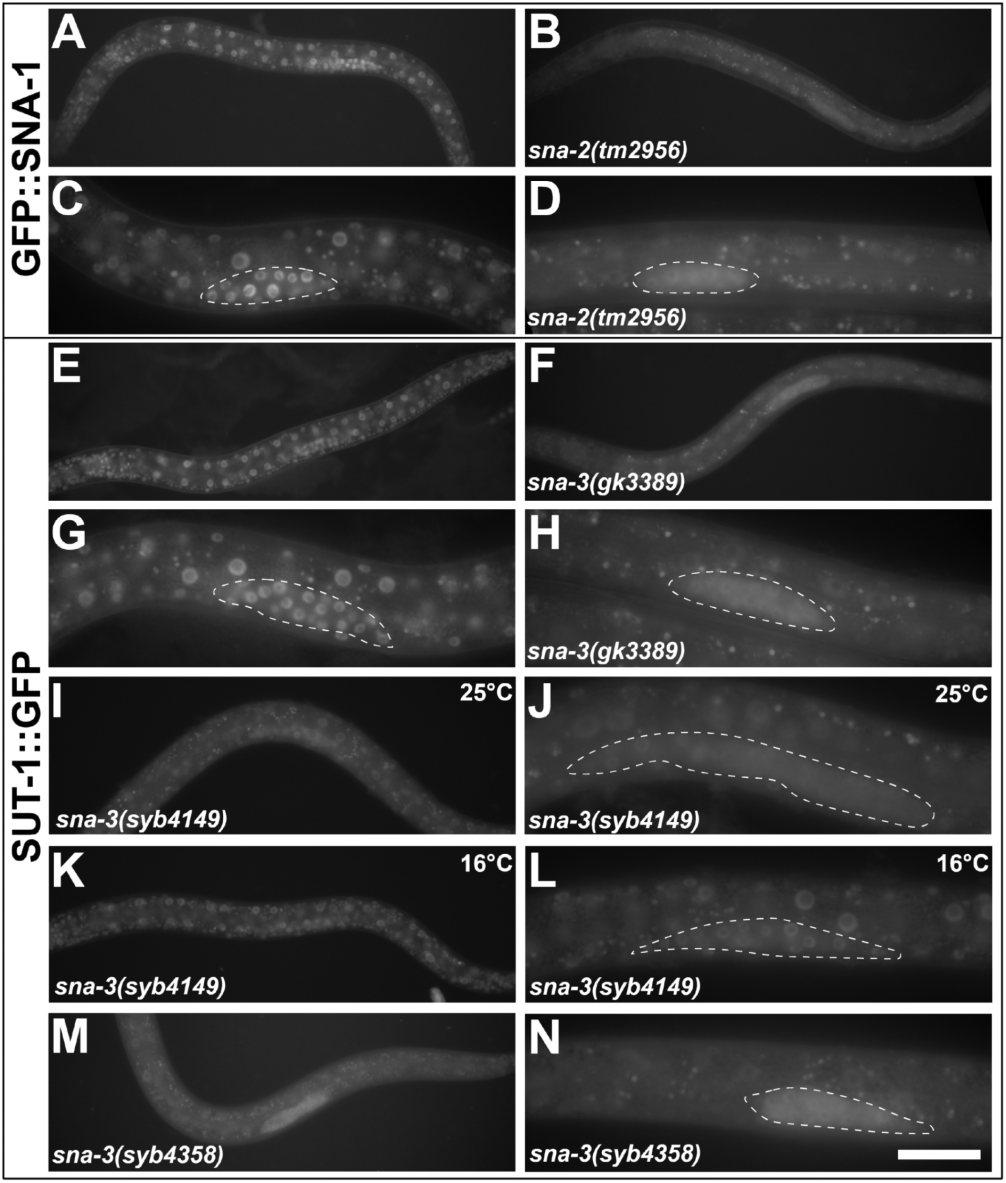
GFP::SNA-1 and SUT-1::GFP nuclear localisation is dependent upon *sna-2* and *sna-3* function, respectively. Representative epifluorescent images of second stage larvae (L2) expressing GFP::SNA-1 (**A–D**) or SUT-1::GFP (**E–N**) in wild type (A, C, E, G), *sna-2(tm2956)* (B, D), *sna-3(gk3389)* (F, H), *sna-3(syb4149)* (**I–L**) and *sna-3(syb4358)* (M, N) homozygotes. Dotted lines indicate the position of the developing germlines in the higher magnification images. Animals were raised at 20°C unless otherwise indicated. Scale bar represents 50 μm (A, B, E, F, I, K, M) or 20 μm (C, D, G, H, J, L, N).

To determine whether the interactions between the proteins observed in Figures 1 and 4 are physiologically relevant, we systematically visualised the localisation of each GFP-tagged protein in different loss-of-function mutant backgrounds (Figure 6 and Supplemental Figure S6). Loss of *sna-2* resulted in significant loss of nuclear localised GFP::SNA-1 (Figure 6B, D) but did not affect the localisation of SUT-1::GFP or SNA-3::GFP (Supplemental Figure S6B, S6D). In contrast, loss of *sna-3* function, using either of the two constitutive loss-of-function mutations, *gk3389* (Figure 6F, H) and *syb4358* (Figure 6M, N) caused reduced levels of nuclear SUT-1::GFP. We saw a similar effect using the temperature-sensitive mutation *syb4149* grown at the restrictive temperature of 25°C (Figure 6I–J), but animals grown at 16°C were much less affected (Figure 6K–L). The sub-cellular distributions of GFP::SNA-1 and SNA-2::GFP were unaffected by loss of *sna-3* function (Supplemental Figure S6F, H).

In all cases, reduction of nuclear localised GFP::SNA-1 or SUT-1::GFP was accompanied by concomitant increases in cytoplasmic fluorescence, showing that SNA-2 and SNA-3, respectively, are essential to retain these proteins in the nucleus, most obviously in the developing germline. We performed Western blots to determine the relative levels of GFP::SNA-1 and SUT-1::GFP in wild type and the respective mutant backgrounds to determine whether some loss of the fluorescence could also be attributed to changes in their steady state levels in response to loss of *sna-2* and *sna-3*, respectively (Supplemental Figure S7). This analysis showed that GFP::SNA-1 levels are reduced by up to two thirds in response to loss of *sna-2* function, and the impact of loss of *sna-3* function on SUT-1::GFP expression levels is even more severe, reducing them to around 20% of wild type levels.

Thus, in addition to (or possibly because of) their depletion from the nucleus in response to the loss of their molecular partners, SNA-1 and SUT-1 steady state levels are significantly impaired. Therefore, the interactions between SNA-2 and SNA-1 and between SNA-3 and SUT-1 that we documented in Figures 1 and 4 have clear functional significance.

### NADAR domain 2 mutations affect SNA-3 expression and localisation

**Figure 7.**
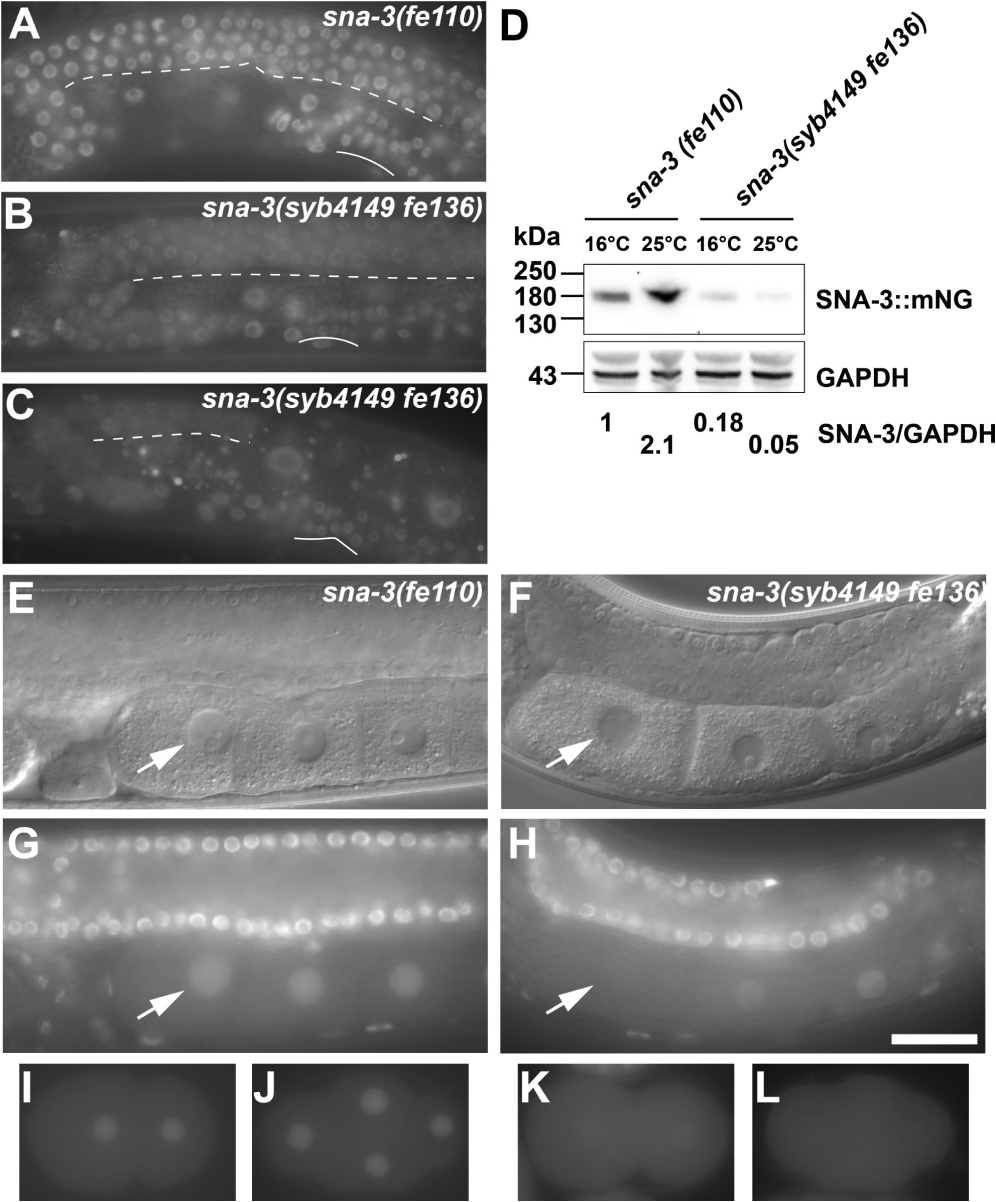
NADAR domain 2 mutations affect SNA-3::mNG expression and localisation. Visualisation of C-terminally-tagged SNA-3 protein expressed from the wild type endogenous coding region (*sna-3(fe110)*; A, G, I, J) or that containing the NADAR domain 2 mutations (*sna-3(syb4149 fe116)*; B, C, F, H, K, **L**). (**A–C**) Representative epifluorescent images of gonad arms from fourth larval stage (L4) hermaphrodites raised at 16°C (A, B) or 25°C (C). Dotted line indicates the location of the ventral surface of the distal gonad arm; unbroken line shows the ventral boundary of the developing uterus. (**D**) Western blots of lysates derived from 500 L4 larvae, raised at the temperatures indicated, probed with anti-mNG and anti-GAPDH antibodies. The expression levels were normalised to the GAPDH loading controls, setting the ratio derived from *sna-3(fe110)* grown at 16°C to unity. (**E, F**) Representative DIC images, and the epifluorescence of the same respective focal planes (**G, H**) of *sna-3(fe110)* (E, G) and *sna-3(syb4149 fe116)* (F, H) adult hermaphrodite gonad arms. Arrows indicate the nucleus of the most mature oocyte in each case. (**I–L**) 2-cell and 4-cell early embryo of *sna-3(fe110)* (**I, J**) and *sna-3(syb4149 fe116)* genotypes (**K, L**). Scale bar represents 20 μm for all panels, except D.

Our data showed that mutation of the three conserved NADAR domain residues in the *sna-3(syb4149)* mutation impairs SNA-3 function. To clarify the effect of these mutations on SNA-3 protein levels and subcellular localisation, we engineered strains to express the endogenous wild type and mutant proteins with a monomeric NeonGreen (mNG) fluorescent tag (17). We visualised expression in the resultant strains that express the mNG tagged wild type protein (*sna-3(fe110*) and four independent *sna-3(syb4149)* mutant strains (*sna-3(syb4149 fe129*), *sna-3(syb4149 fe131*), *sna-3(syb4149 fe135*) and *sna-3(syb4149 fe136*)) (Figure 7). The expression and nuclear localisation of SNA-3::mNG (Figure 7A,G) was the same as we saw for SNA-3::GFP (Figure 5). All four mNG-tagged *syb4149* mutant strains displayed the same nuclear localisation in somatic cells at both restrictive and permissive temperatures (see, for example, the developing uterine cells in Figure 7B and C) but at greatly reduced levels compared to mNG tagged wild type SNA-3 (Figure 7D). At the restrictive temperature, fluorescence is also weakly detected in the cytoplasm in the germline (Figure 7C), suggesting that the mutation has a stronger impact on SNA-3 activity in the germline, which is consistent with the sterile phenotype of animals raised at the restrictive temperature (Figure 3D).

One striking difference between the wild type and mutant SNA-3::mNG localisation patterns is a loss of nuclear fluorescence during oocyte maturation and early zygotic development in *syb4149* mutants (Figure 7G–L). It is known that during oogenesis transcription shuts down on entry into diakinesis and transcription resumes during early embryogenesis (45,46). It is thus likely that the loss of SNA-3::mNG we observe is a consequence of decreased steady-state levels in *syb4149* mutants leading to gradual loss of protein once global gene expression is switched off. Restoration of nuclear fluorescence coincides with activation of zygotic gene expression in the major cell lineages of the early embryo.

### SNA-3 links the SL1 snRNP to conserved molecular complexes active at 5′ RNA cap

**Figure 8.**
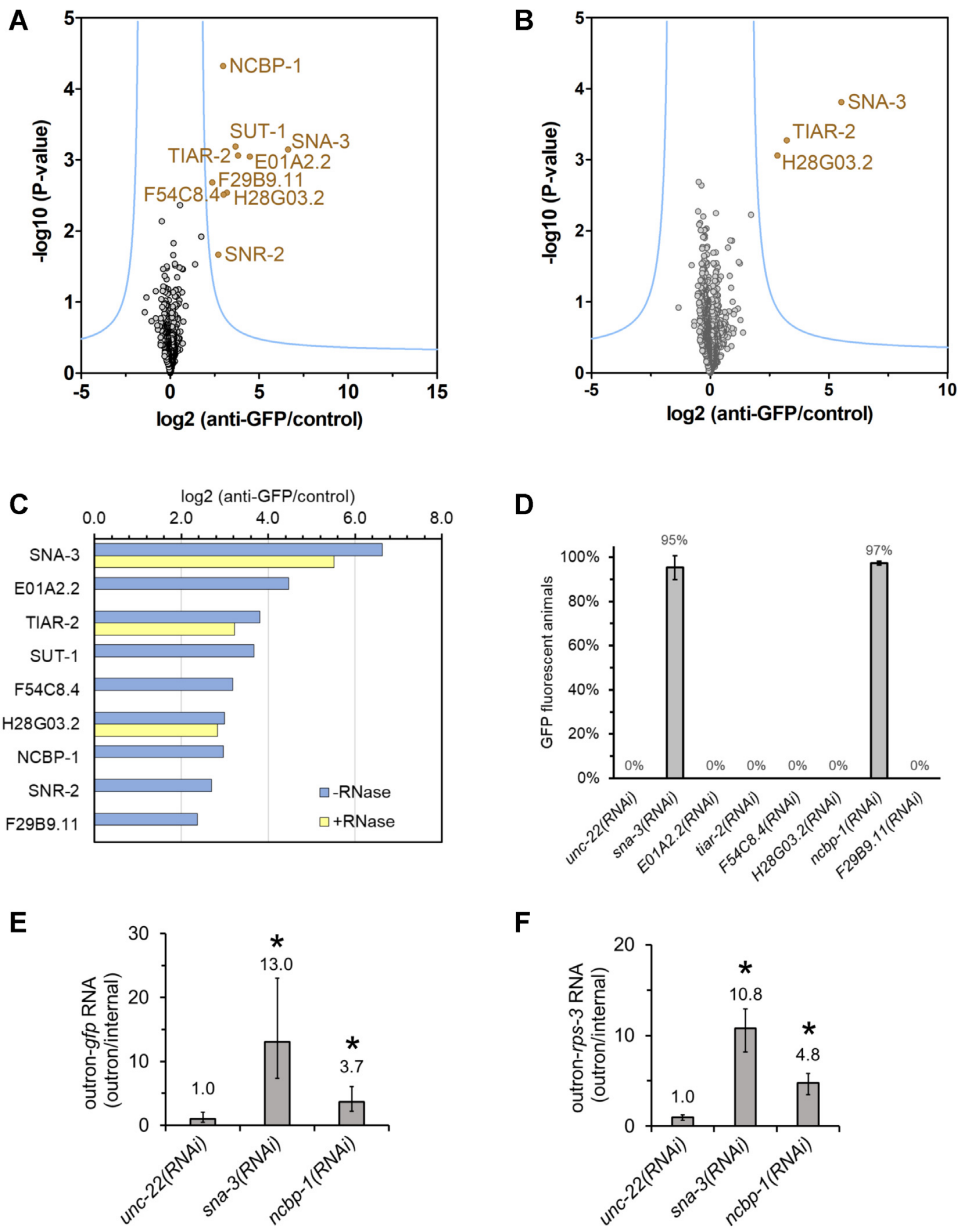
SNA-3 interacts with the CBC-ARS2 complex and other proteins involved in mRNA processing. Immunoprecipitations and control reactions were done in triplicates using embryonic extract from PE975 animals expressing GFP-tagged SNA-3 treated without/with RNase A/T1 and analysed as described in Figure 1 legend. (**A**) Volcano plot showing the proteins interacting with SNA-3 identified by co-immunoprecipitation with GFP::SNA-3 from embryonic extract. Proteins in gold are significantly enriched (false discovery rate **≤** 0.01 and S0 of 2.5). (**B**) Volcano plot showing proteins interacting with SNA-3 in an RNA-independent manner by immunoprecipitation from extract pre-treated with RNases. (**C**) Proteins enriched in immunoprecipitation from extract treated without/with RNase A/T1 are shown in blue and yellow, respectively. The fold enrichment in SNA-3 immunoprecipitations is plotted on the X-axis (log_2_ anti-GFP/control). Normally, proteins are identified by abbreviations based on their gene names, or if the gene has not been named, the open reading frame identifier. Note that SNA-3, H28G03.2 and F29B9.11 only appear with their Uniprot identifiers Q9GYR5, H2L0D2 and Q9GYI1, respectively, in Supplemental File 3. (D–F) Identification of new factors required for SL1 *trans*-splicing by RNAi knockdown. The effect of RNAi knockdown on SL1 *trans*-splicing was analysed using the GFP expression in PE793 animals carrying the P*vit-2*::outron::GFP^M1A^ transgene subjected to RNAi treatments by fluorescent microscopy and using RT-qPCR assays described in Figure 2. (**D**) The proportion of GFP fluorescent animals was determined by fluorescence microscopy. Error bars represent the standard deviation based on three independent experiments. (**E, F**) Detection of SL1 *trans*-splicing inhibition of *gfp* and r*ps-3* transcripts by RT-qPCR. The analysis was done as described in Figure 2 legend. Error bars show standard deviation from three technical replicates. * indicates treatments where SL1 *trans*-splicing is significantly inhibited compared to *unc-22(RNAi)* (t-test, p≤ 0.05). Similar results were obtained in two independent experiments.

To try to understand the role played by SNA-3 in SL1 *trans*-splicing, we analysed the proteins that interact with GFP-tagged SNA-3 using the same immunoprecipitation strategy outlined above (Figure 8 and Supplemental File 3). We identified 8 proteins that were significantly enriched in GFP::SNA-3 immunoprecipitations from embryonic extracts compared to controls using the strategy outlined above.

Among these proteins was SUT-1, providing independent confirmation of the interactions between this protein and SNA-3 shown in Figures 4 and 6. Surprisingly, the SNA-3 - SUT-1 immunoprecipitation interaction is RNA-dependent, which might lead one to conclude that it is indirect. However, it is possible that the SNA-3 - SUT-1 interaction detected by yeast two-hybrid assay may need to be stabilised by the presence of RNA under physiological conditions.

**Figure 9.**
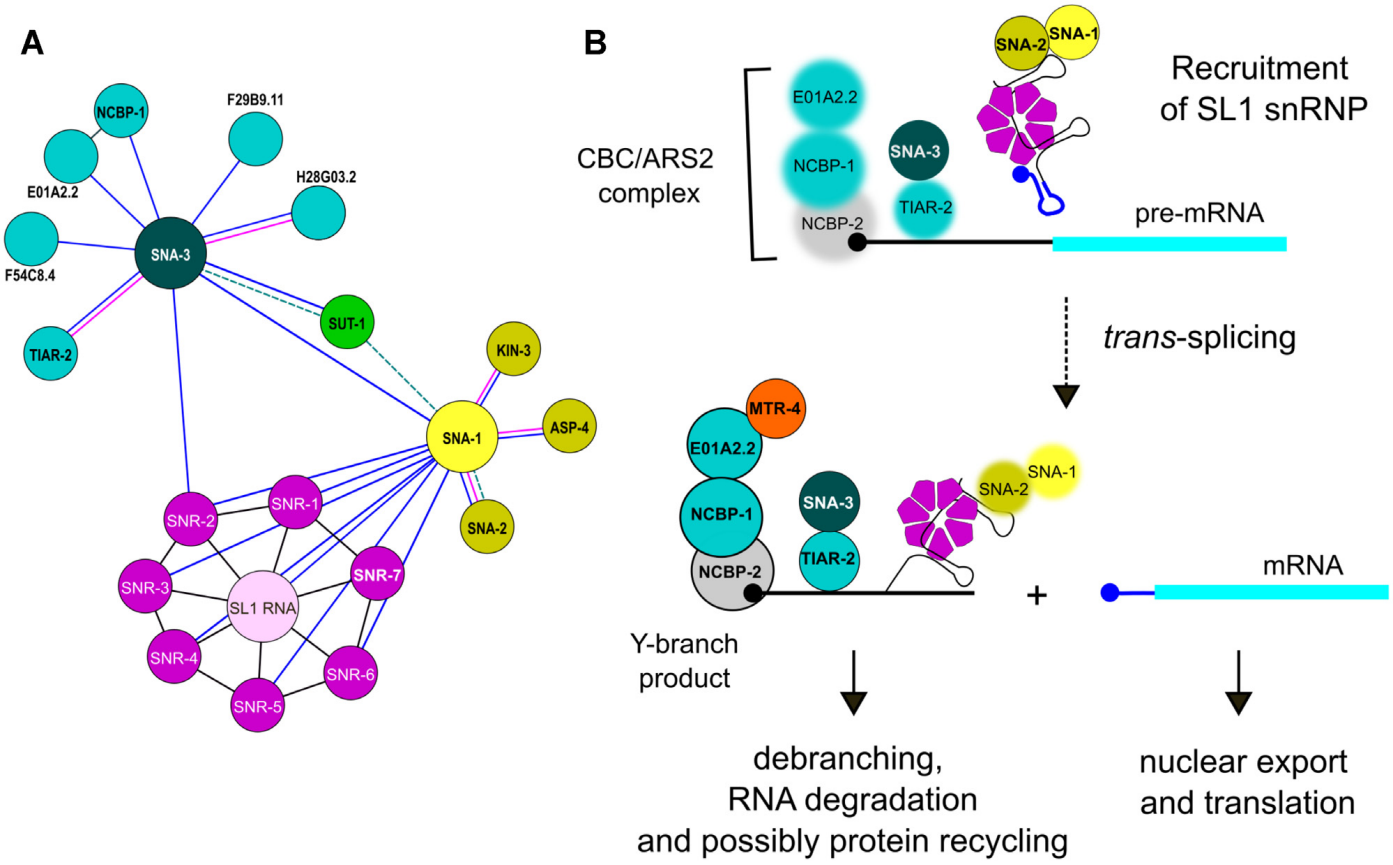
SNA-3 is a key factor in an RNA-protein interaction network connecting the SL1 snRNP with the CBC-ARS2 complex. (**A**) The network shows interactions between the SL1 snRNP (with components SL1 RNA, SNA-1, SNA-2 and Sm proteins) and SNA-3 and associated proteins, including SUT-1 and the CBC-ARS2 complex components NCBP-1 and E01A2.2. Significant interactions detected in immunoprecipitations using embryonic extracts treated without/with RNase are shown in blue and purple, respectively (Figures 1A, B; 8A, B). Interactions detected by yeast two-hybrid assay are shown as dashed green lines (Figure 4). Interactions between Sm proteins and SL1 RNA and between NCBP-1 and E01A2.2 are based on published data and are shown in black (60, 61). Networks were drawn using Gephi (62). (**B**) Model for the role of SNA-3 in SL1 *trans*-splicing. SNA-3 may coordinate spliced leader *trans*-splicing with transcription by mediating the recruitment of the SL1 snRNP to pre-mRNA bound by the CBC-ARS2 complex, and also act later during processing of the Y-branch product of the *trans*-splicing reaction. The SL1 snRNP may be recruited to the pre-mRNA outron through interactions involving TIAR-2 associated with U-rich outron regions, and SNA-3. Once *trans*-splicing has occurred, the mRNA with the SL1 spliced leader (dark blue) at the 5′ end is exported and translated. The Y-branch product, composed of the remnant of the SL RNA (black) connected to the outron RNA by a 2′-5′ phosphodiester bond at the branch point, and bound by the CBC-ARS2 complex and MTR-4, SNA-3 and TIAR-2, Sm proteins and possibly SNA-1 and SNA-2 proteins, is processed and degraded. SNA-3 may participate in this processing, with the CBC-ARS2 complex and MTR-4 detected in SNA-3 immunoprecipitations (Figure 8A, Supplemental File 3), targeting the RNAs to the NEXT complex for degradation. Protein-protein and protein-RNA contacts are based on (A), and contacts between RNAs and TIAR-2 and SNA-2 are hypothetical. The CBC-ARS2 components E01A2.1 and NCBP-1, and TIAR-2 when associated with pre-mRNA are shown blurred, indicating they were not detected in GFP::SNA-1 immunoprecipitations. SNA-1 and SNA-2 associated with the Y-branch product are shown blurred, reflecting they are not detected in immunoprecipitations of SNA-3::GFP. NCBP-2 is shown in grey. For clarity, only selected factors shown in A are included in B.

We detected enrichment of SNR-2/SmB in SNA-3 immunoprecipitates, which might be expected for a protein associated with SL1 snRNP. The other proteins we identified conferred more insight into SNA-3 function and are discussed below and shown in Figure 9A.

NCBP-1 and E01A2.2 are *C. elegans* orthologues of components of the Cap Binding Complex (CBC)-ARS2 ternary complex that is involved in context-dependent RNA processing events. The second component of the cap binding complex, NCBP-2, has a weaker interaction with SNA-3: it was enriched in two out of three immunoprecipitations using anti-GFP nanobodies but was not detected in any of the control precipitations. As we insisted on proteins needing to be present in all three immunoprecipitations, NCBP-2 is not included in the analysis shown in Figure 8A.

F54C8.4 is one of three *C. elegans* homologues (47,48) of dual specificity phosphatases that act to dephosphorylate the 5′ ends of RNAs. Interaction of SNA-3 with these proteins is consistent with a role in facilitating the association between SL1 snRNP and the 5′ end of the target pre-mRNAs, both during and following the *trans*-splicing reaction.

TIAR-2 is a homologue of the mammalian RNA-binding proteins, TIA-1/TIAR. These proteins, which are implicated in the formation of stress granules, are selective RNA binding proteins, and TIA-1 is involved in splice site recognition (49). Thus, TIAR-2 may have a similar role in *C. elegans* in spliced leader *trans*-splicing, explaining its association with SL1 RNA, either because it binds to a U-rich sequence in the SL1 RNA and/or in the outron of the target pre-mRNA. The interaction between SNA-3 and TIAR-2 is resistant to pre-treatment of extract with RNases, suggestive of a direct protein-protein interaction (Figure 8B, C).

The significance of the remaining SNA-3 interactors is harder to evaluate. F29B9.11 and H28G03.2 are proteins without known functions, and without homologues outside of the nematodes. H28G03.2 is the only other protein (along with TIAR-2) from our immunoprecipitations whose SNA-3 interaction is insensitive to RNase treatment.

We tested the functional significance of the protein interactions with SNA-3 by asking whether their loss of function (using RNAi) had any effect on the SL1 *trans*-splicing reporter that we used above. We have shown earlier that knockdown of *sut-1* expression has a small but clear inhibitory effect on SL1 *trans*-splicing (9). Of the other SNA-3 interacting proteins, only RNAi knockdown of NCBP-1 resulted in activation of the *gfp* reporter gene, indicating an inhibition of SL1 *trans*-splicing (Figure 8D). We were able to confirm this using qPCR to detect an inhibition of SL1 *trans*-splicing of *gfp* and *rps-3* transcripts (Figure 8E, F).

The CBC–ARS2 complex binds to the cap structure at the 5′ end of RNAs and acts as a platform for the co-transcriptional recruitment of other factors that determine transcript fate (50). We searched for such factors among the proteins enriched in SNA-3::GFP immunoprecipitations, using a lower threshold of enrichment (*q*-value ≤ 0.15). We detected near 2-fold enrichment of only one known CBC-ARS2 factor, MTR-4, an RNA helicase that acts in the nuclear NEXT and PAXT RNA decay pathways recruited by the CBC-ARS2 complex to RNA. The RNA-dependent interaction of the CBC-ARS2 complex with SNA-3 is consistent with a role for SNA-3 in coordinating SL1 *trans*-splicing with the transcription of the target *trans*-spliced pre-mRNA, promoting interaction with pre-mRNA, possibly through TIAR-2, but it would also be well placed to facilitate the processing of the Y-branch product via MTR-4-mediated recruitment to the nuclear exosome (Figure 9B).

## DISCUSSION

We have carried out the first comprehensive molecular and functional characterisation of the RNA and protein components of the *C. elegans* SL1 snRNP. This work complements and significantly extends the seminal studies that first elucidated the nature of the nematode spliced leader *trans*-splicing machinery (2–5,7,51,52). Importantly, we have established the functional significance of the interactions that we identified through assaying the effect of loss of function of each component on spliced leader *trans*-splicing. This work has extended the number of components known to be involved in this process, adding molecules with known biochemical functions, as well as new constituents with previously undefined functions. It has also elucidated critical interactions between known components of the spliceosome and the SL1 snRNP; and confirmed previously suspected elements of the latter complex.

We have confirmed that the SL1 RNA forms an snRNP distinct to the SL2 snRNP: we do not detect SL2 RNA in GFP::SNA-1 immunoprecipitations (Table 1), supporting previous studies which showed that SNA-1 does not associate with SL2 RNA (8). Similarly, we see mild enrichment in the GFP::SNA-1 immunoprecipitations of one of the family of twelve enigmatic SmY RNAs. Again, this is consistent with previous studies showing that this class of nematode-specific RNAs are associated with spliced leader *tran*s-splicing (3,8). Intriguingly, we also see several protein-coding transcripts (from genes whose mRNAs are normally SL1 *trans*-spliced) that are similarly enriched. Since these transcripts retain their nascent 5′ UTRs, this suggests that the GFP::SNA-1 pull-down is capturing complexes actively engaged in SL1 *trans*-splicing. We also observed 3.6-fold enrichment of the snoRNA encoded by ZK185.9, but the significance of this is unclear - ZK185.9 is not conserved in other nematodes, even in close relatives, so it is unlikely to represent a component of the SL1 *trans*-splicing mechanism.

The most enriched proteins in the GFP::SNA-1 pull-downs were also those that were the least surprising to see, since they are previously described constituents of the *C. elegans* SL1 snRNP: SNA-2, SNA-1 and the seven Sm/SNR proteins (Figures 1 and 9A) (5,8,9). The interaction between SNA-1 and SNA-2 is most likely a direct protein-protein interaction as it is not dependent on RNA and can be reproduced in yeast two hybrid assays (Figures 1 and 4). We have verified the functional significance of this interaction by showing that in the absence of SNA-2, SNA-1 is no longer enriched in the nucleus and is found at high levels in the cytoplasm (Figure 6) and its steady state levels are reduced (Supplemental Figure S7).

There is one notably absent protein that we expected to recover based on previous work: Denker and colleagues (7) identified the splicing factor SF1 as a SNA-1 interacting protein in *Ascaris* extracts, but this protein was absent from GFP::SNA-1 immunoprecipitations. It's possible that this is not a robust enough interaction to survive the conditions that we used.

The other enriched proteins are functionally diverse. There are those with known roles in RNA splicing, such as the spliceosome components, PRP-8 and PRP-19, which are components of the catalytically active spliceosome (reviewed in (53,54); and SMN-1, which in addition to its role in snRNP biogenesis, also plays an important role in the association of snRNPs with pre-mRNA (40,41). These data extend our knowledge of how SL1 RNA interacts with the spliceosome, showing that it interacts much the same way as the pre-mRNA 5′ splice site in *cis*-splicing. This is consistent with what we expect to see in spliced leader *trans*-splicing. The identification of GFP::SNA-1 interacting proteins with known functions but no direct functional links to RNA splicing is harder to interpret without further investigation of each interacting protein. There may be clues amongst these for novel activities shaped by the specific requirements of spliced leader *trans*-splicing, such as the need to recover Sm proteins from Y-branch products. One intriguing set of interactions, the recovery of alpha- and beta-tubulin subunits suggests that SL1 snRNP interacts with microtubules and indeed we see transient associations between mitotic spindles and GFP::SNR-2/SmB (see Supplemental Movie 1) in early embryos that perhaps explains this observation.

Many of the GFP::SNA-1 interacting molecules whose functions in SL1 *trans*-splicing we tested gave negative results (Figure 2). We observed similar distributions of negative signals for the GFP::SNA-3 interactors (Figure 8). It is conceivable that these are caused by a concomitant inhibition of *cis*-splicing of the *gfp* reporter gene transcript necessary for GFP expression. Alternatively, the gene knockdown by RNAi may not have resulted in a sufficient reduction of protein to cause an inhibition of SL1 *trans*-splicing.

The identification of SNA-3 adds a new, previously uncharacterised protein to the nematode SL1 *trans*-splicing machinery. Importantly, this protein is found in all nematode species for which we have genome sequence information, but is absent outside the phylum, consistent with a protein that has been recruited into a function specific to spliced leader *trans*-splicing. Understanding the function of this protein will thus provide important insights into the cellular mechanism needed to perform spliced leader *trans*-splicing.

Inferring its role based on what we know about NADAR domains from the limited number of studies of bacterial and plant family members is challenging; the NADAR domain is rarely found in animal proteins and is entirely absent from vertebrates. A functional overview of the domain, based on bacterial operon and multidomain protein contexts, indicates that it tends to be associated with RNA processing events that use NAD and/or generate ADP-ribose derivatives (42). Direct evidence for the role of the domain comes from its association with riboflavin biosynthesis enzymes in bacteria and plants, where it clearly functions as an N-glycosidase (43). Although further work is required to identify the biochemical roles of each SNA-3 NADAR domain (together with their likely substrate(s)), our genetic analysis shows that individual NADAR domains play critical roles in SNA-3 function (Figure 3). This work also shows that mutation of residues homologous to those important for N-glycosidase function in bacteria and plants impairs SNA-3 function. This raises the possibility that the NADAR domains in SNA-3 have a similar function. Of course, it may also be that our mutations perturb the overall folding of the affected domain and thus impact SNA-3 function indirectly. Our analysis of the expression and localisation of the mNG-tagged NADAR domain 2 mutant protein shows that even at the restrictive temperature nuclear localisation is largely unaffected in somatic cells (Figure 7). This indicates that the mutations don’t impact the overall folding of the protein, at least for a significant proportion of the SNA-3 molecules. However, even at the permissive temperature, levels of SNA-3 are reduced in this mutant, and this reduction is further enhanced at the restrictive temperature, possibly a reflection of their impaired interactions created by loss of a functional NADAR domain 2.

Further hints about SNA-3 function come from the interactions we detected in SNA-3::GFP immunoprecipitates. In particular, the interaction of SNA-3 with the CBC-ARS2 complex detected is consistent with an association of SNA-3 with the 5′ outron of the pre-mRNA undergoing spliced leader *trans*-splicing (Figure 8). Specifically, we have detected RNA-dependent interactions with the components of the CBC-ARS2 complex, NCBP-1 and E01A2.2, and the putative 5′ polyphosphatase, F54C8.4, which, based on homology, would be predicted to act at the 5′ end of the pre-mRNA.

We rule out an alternative hypothesis, that the proteins act at the 5′ end of the nascent SL1 RNA, because SNA-3 clearly interacts with Sm protein-loaded SL1 RNA, which does not occur until after the SL RNA has been assembled into an snRNP and imported into the nucleus. Furthermore, the RNA-independent interaction with TIAR-2, a protein whose mammalian homologue, TIA-1, influences 5′ splice-site choice by binding to U-rich intron sequences and interacting with the U1 snRNP C protein (49,55,56), suggests that TIAR-2 may interact with U-rich sequences in either the SL1 RNA or the outron of the pre-RNA, perhaps facilitating the recruitment of the SL1 snRNP through its interaction with SNA-3 (Figure 9B).

The association between SNA-3 and the CBC-ARS2 complex, and enrichment of MTR-4 in SNA-3::GFP pull-downs, suggests another, not mutually exclusive, function in the processing of the Y-branch product that is produced by every SL1 *trans*-splicing event. Unlike its *cis*-splicing analogue, the lariat, the Y-branch RNA contains a 5′ cap structure, and is associated with the Sm proteins, of which the latter must be either degraded or recycled. In either scenario, there is likely a need for novel biochemical activity to process the Sm proteins and degrade the Y-branch. Through its interactions with the Y-branch product bound by the CBC-ARS2-MTR-4 complex, SNA-3 would be well placed to facilitate the processing of this by-product of the *trans*-splicing reaction, leading ultimately to degradation by the nuclear exosome (Figure 9B).

SNA-3 also interacts with SUT-1, a protein previously implicated in spliced leader *trans*-splicing (8). We detected interactions between SNA-3 and SUT-1 using two independent approaches: yeast two-hybrid assays (Figure 4) and co-immunoprecipitations (Figure 8). In addition, both the steady-state levels and subcellular localisation of SUT-1::GFP are SNA-3-dependent (Figure 6; Supplemental Figure S7). This implies that SUT-1 through its interactions with SNA-3, interacts with the SL1 *trans*-splicing machinery. This is surprising since previous work suggests that SUT-1 forms a distinct, SNA-2-containing complex that does not contain SL1 RNA (8).

However, our yeast two-hybrid analysis failed to detect any direct interaction between SUT-1 and SNA-2, and the only region of sequence similarity shared by SNA-1 and SUT-1, a region that was previously proposed to be the SNA-2 interaction motif (8), is not required for SNA-1’s interactions with SNA-2 (Figure 4; Supplemental Figure S4). Rather, the region of SNA-1 involved in SNA-2 binding appears to be absent from SUT-1, further emphasising their lack of interaction.

How might we account for the apparent contradictions between the two studies? Although SNA-3 was found in GFP::SNA-1 pull-downs, the converse is not the case. Moreover, aside from the presence of the SNR/Sm proteins, the two sets of immunoprecipitations did not identify the same set of interactors. We might expect that the GFP::SNA-1 and SNA-3::GFP immunoprecipitations capture the steady-state levels of the complexes in which the two proteins spend most of their respective times. This could explain why at least some GFP::SNA-1 complexes contain SNA-3, while few or none of the SNA-3::GFP complexes are associated with SNA-1. This is compatible with a role for SNA-3 in *trans*-splicing independent of SNA-1, and favours SNA-3 acting late in *trans*-splicing such as in processing of the Y-branch product (Figure 9).

According to such a model, one might expect to be able to detect interactions between SUT-1 and SNA-2 through immunoprecipitation experiments, explaining the data of MacMorris and colleagues (8). Though why they were able to detect these interactions while we have not isn’t immediately clear. Perhaps, this stems from the different conditions under which immunoprecipitations were performed; we supplemented our embryonic extracts with MgCl_2,_ which is required for the catalysis of RNA splicing, MacMorris and colleagues detected interactions between SNA-2 and SUT-1 in the presence of EDTA (8). Ultimately, determining the validity of this interpretation, as well as the models outlined above, will require detailed biochemical and structural studies of the individual proteins and RNAs and their interactions; the work set out in this paper provides the framework to guide these future investigations.

## DATA AVAILABILITY

RIP-Seq data have been deposited in the ArrayExpress database at EMBL-EBI (www.ebi.ac.uk/arrayexpress) (57) under accession number E-MTAB-10289 (see also Supplemental File 2).

The mass spectrometry proteomics data have been deposited to the ProteomeXchange Consortium via the PRIDE (58) partner repository with the dataset identifier PXD024763 (see also Supplemental File 3).

## Supplementary Material

gkac534_Supplemental_Files

## Notes

Present address: Rotimi Yemi Fasimoye, MRC Protein Phosphorylation & Ubiquitylation Unit, Sir James Black Centre, University of Dundee, Dundee DD1 5EH, Scotland, UK.

Present address: Sarah Brivio, School of Medicine, Department of Pathology and Immunity, University of Geneva, Centre Médical Universitaire, Rue Michel-Servet 1, Geneva 1206, Switzerland.

Present address: Carolina Mangana, CeMM Research Centre for Molecular Medicine of the Austrian Academy of Sciences, Lazarettgasse 14, AKH BT 25.3, 1090 Vienna, Austria.

Present address: Marius Wenzel, School of Biological Sciences, University of Aberdeen, Aberdeen AB24 2TZ, Scotland, UK.
