# Supplementary material for: A novel, essential trans-splicing protein connects the nematode SL1 snRNP to the CBC-ARS2 complex
Source: Nucleic Acids Res. 2022 Jun 23;50(13):7591–607. doi: 10.1093/nar/gkac534 (PMC9303266; doi:10.1093/nar/gkac534)
Supplement: gkac534_Supplemental_Files [file gkac534_supplemental_files.zip › Captions for supplemental files and movie.docx]

**Supplemental File 1: RNA and DNA sequences used for molecular cloning and CRISPR mediated genome engineering.** The spreadsheet includes oligonucleotide sequences used for the preparation of (i) RNAi feeding vectors, (ii) yeast two-hybrid plasmids and (iii) constructs for GFP and mNeonGreen tagging by genome engineering. For the preparation of RNAi feeding vectors and yeast two-hybrid plasmids, target sequences were PCR amplified from genomic DNA or cDNA and inserted by In-Fusion cloning into the plasmid pPD129.36 (for RNA interference) or into pGBKT7 or pGADT7 (for yeast two-hybrid assays) as described in Material and Methods. Guide RNA constructs and homology repair templates for genome engineering were prepared as described in Material and Methods.

**Supplemental File 2: Identification of RNAs associated with SNA-1 by RIP-Seq analysis.** The spreadsheet contains the full results of the RIP-Seq data shown in Table 1. Immunoprecipitations from PE906 embryonic extracts, RNA extractions, sequencing and data analysis were done as described in Table 1 legend and Material and Methods.

**Supplemental File 3: Identification and quantification of proteins interacting with SNA-1 and SNA-3 by immunoprecipitation followed by mass-spectrometry.** The spreadsheet contains the full results of the analysis of immunoprecipitations done with embryonic extracts prepared from PE906 and PE975 and N2 animals shown in Figure 1, Figure 7 and Supplemental Figure 1. Immunoprecipitation, protein identification and quantification were done as described in Figure legends and Materials and Methods. SNA-3, H28G03.2, F29B9.11 and PRDX-2 only appear with their Uniprot identifiers Q9GYR5, H2L0D2, Q9GYI1 and PRDX-2, respectively.

**Supplemental Movie 1: GFP::SNR-2/SmB expression in an early embryo**

The movie shows a time-lapse series of single focal plane confocal images. The exposure was set to more easily visualise the cytoplasmic and spindle associated GFP fluorescence (arrows indicating anaphase spindles). Note the rapid relocalisation of fluorescence as the nuclear membrane breaks down and reforms.
