# Supplementary material for: A novel, essential trans-splicing protein connects the nematode SL1 snRNP to the CBC-ARS2 complex
Source: Nucleic Acids Res. 2022 Jun 23;50(13):7591–607. doi: 10.1093/nar/gkac534 (PMC9303266; doi:10.1093/nar/gkac534)
Supplement: gkac534_Supplemental_Files [file gkac534_supplemental_files.zip › Fasimoye et al Supplemental Figures.pdf]

Fasimoye *et al,* Legends to Supplemental Figures

Supplemental Figure 1. Proteome analysis of immunoprecipitations performed with embryonic extracts prepared from N2 animals. The volcano plot was prepared using the enrichment in anti-GFP nanobody immunoprecipitations compared to controls (log2 (GFP/control), and the false discovery rate (-log10 (P-value). The significance cutoff curve is drawn in blue (FDR **<** 0.01 and S0 of 2.5).

Supplemental Figure 2. RNase treatment of embryonic extracts. Shown is RNA isolated from embryonic extract treated without (-) or with (+) 200 units/ml RNase T1 and 5 units/ml RNase A. RNase treatment was for 1h at 4°C. RNA was isolated by treating 4µl extract with 16 µl of 2.5 µg/µl Proteinase K, 0.6% SDS, 50 mM EDTA for 15 min at 37°C and then analysed by agarose gel electrophoresis and visualised by staining with ethidium bromide. M is the molecular weight marker.

Supplemental Figure 3. Phylogeny of individual SNA-3 NADAR domains. The phylogeny was created from a MUSCLE alignment (1) of individual SNA-3 NADAR domains from selected nematodes, using functionally characterised non-nematode NADAR domains as an outgroup (Supplemental File 5). The phylogeny was created using W-IQ-TREE (2) (default settings, autodetection of substitution model; ultrafast bootstrap analysis on 1000 bootstrap alignments). The resultant Newick tree was visualised and prepared for publication using FigTree v1.4.4 (https://github.com/rambaut/figtree/). Support values shown for each node are ultrafast bootstrap support values (%). Species abbreviations: Asu, *Ascaris suum*; Ana, *Acrobeloides nanus*; Bma, *Brugia malayi*; Bxy, *Bursaphelenchus xylophilus*; Cel, *C. elegans*; Cin, *Caenorhabditis inopinata*; Dim, *Dirofilaria immitis*; Gpa, *Globodera pallida*; Hgl, *Heterodera glycines*; Hme, *Halicephalobus mephisto*; Oti, *Oscheius tipulae*; Pre, *Panagrellus redivivus*; Psa, *Plectus sambesii*; Sba, *Soboliphyme baturini*; Tmu, *Trichuris muris*; Tsp, *Trichinella spiralis*.

Supplemental Figure 4. Interaction of SNA-1 with SNA-2 and of SUT-1 with SNA-3 does not require the N-terminal region conserved between SNA-1 and SUT-1. Analysis of protein-protein interactions between SNA-1 and SNA2, and SUT-1 and SNA-3. Top: AlphaFold predictions for SNA-1 and SUT-1, only showing residues 1 – 60, which correspond to the regions of high sequence similarity between the two proteins (3). Red shading indicates the regions deleted in the respective yeast two-hybrid constructs. MUSCLE alignment (1) of SNA-1 and SUT-1 orthologues. Species abbreviations: Asu, *Ascaris suum*; Bma, *Brugia malayi*; Cbr, *Caenorhabditis briggsae*; Cel, *C. elegans*; Hco, *Haemonchus contortus*; Llo,

Loa loa. Bottom: The conserved regions between amino acids 16 and 50 of SNA-1 or SUT-1 were removed from pGADT7 plasmids expressing full-length SUT-1 or SNA-1 by Q5 site-directed mutagenesis (New England Biolabs) using the primers listed in Supplemental File 1. Sequences were confirmed by DNA sequencing (Eurofins Genomics). Interactions were detected by expressing SNA-2 and SNA-3 as Gal4 DNA-binding domain fusion proteins (DB) using pGBKT7 derivatives in Y2HGold yeast, and SNA-1, SNA-1Δaa16-50, SUT-1 and SUT-1Δaa16-50 as Gal4 activation domain fusion proteins (AD) from pGADT7 derivatives in Y187 yeast. No insert controls were done with unmodified pGBKT7 or pGADT7. Diploids were plated as controls on synthetic defined without leucine or tryptophan (-Leu -Trp) and on synthetic defined medium without adenine, histidine, leucine and tryptophan (-Ade -His -Leu-Trp) to test for the activation of the Ade2 and His3 reporter genes.

Supplemental Figure 5. The SNA and SUT-1 GFP fusion proteins are localised to the nucleus of all germline and somatic cells. Images show anaesthetised adult hermaphrodites, focussed on the anterior (GFP::SNA-1 and SUT-1::GFP) or posterior (SNA-2::GFP, SNA-3::GFP and SNR-2/SmB::GFP) gonad arms. SNR-2/SmB is shown as a control - SmB proteins are primarily localised to the nucleus in eukaryotes. Exposure times are given in milliseconds (ms). Scale bar indicates 25 micrometres.

Supplemental Figure 6. SUT-1::GFP/SNA-3::GFP and SNA-2::GFP/GFP::SNA-1 remain nuclear localised in loss of *sna-2* and *sna-3* function backgrounds, respectively. Representative epifluorescent images of second stage larvae (L2) expressing the indicated fluorescently tagged protein in wild type (A, C, E, G), *sna-2(tm2956)* (B, D) or *sna-3(gk3389)* (F, H) homozygotes. Dotted lines delineate the developing germline primordium. Scale bar represents 20 μm.

Supplemental Figure 7. GFP::SNA-1 and SUT-1::GFP levels are reduced in loss of *sna-2* and *sna-3* function backgrounds, respectively. Western blot analysis comparing GFP::SNA-1 levels in wild type (+) and *sna-2(tm2956)* homozygotes, and SUT-1::GFP levels in wild type (+) and *sna-3(gk3389)* homozygotes. (A) Top panels: anti-GFP antibodies detected GFP::SNA-1 and SUT-1::GFP in 400 and 200 second stage larvae (L2), respectively. Bottom panels: section of membranes stained with amido black to visualise total proteins. (B) The graph shows levels of GFP::SNA-1 and SUT-1::GFP proteins in *sna-2(tm2956)* and *sna-3(gk3389)* animals relative to wild type. Protein levels were standardised using amido black staining as reference, and GFP protein levels in wild type animals were defined as unity. Data shown are from 2 independent experiments.

REFERENCES

1. Edgar,R.C. (2004) MUSCLE: multiple sequence alignment with high accuracy and high throughput. *Nucleic Acids Res.*, **32**, 1792–1797.

2. Trifinopoulos,J., Nguyen,L.-T., von Haeseler,A. and Minh,B.Q. (2016) W-IQ-TREE: a fast online phylogenetic tool for maximum likelihood analysis. *Nucleic Acids Res.*, **44**, W232–5.

3. Jumper,J., Evans,R., Pritzel,A., Green,T., Figurnov,M., Ronneberger,O., Tunyasuvunakool,K., Bates,R., Žídek,A., Potapenko,A., *et al.* (2021) Highly accurate protein structure prediction with AlphaFold. *Nature*, 10.1038/s41586-021-03819-2.

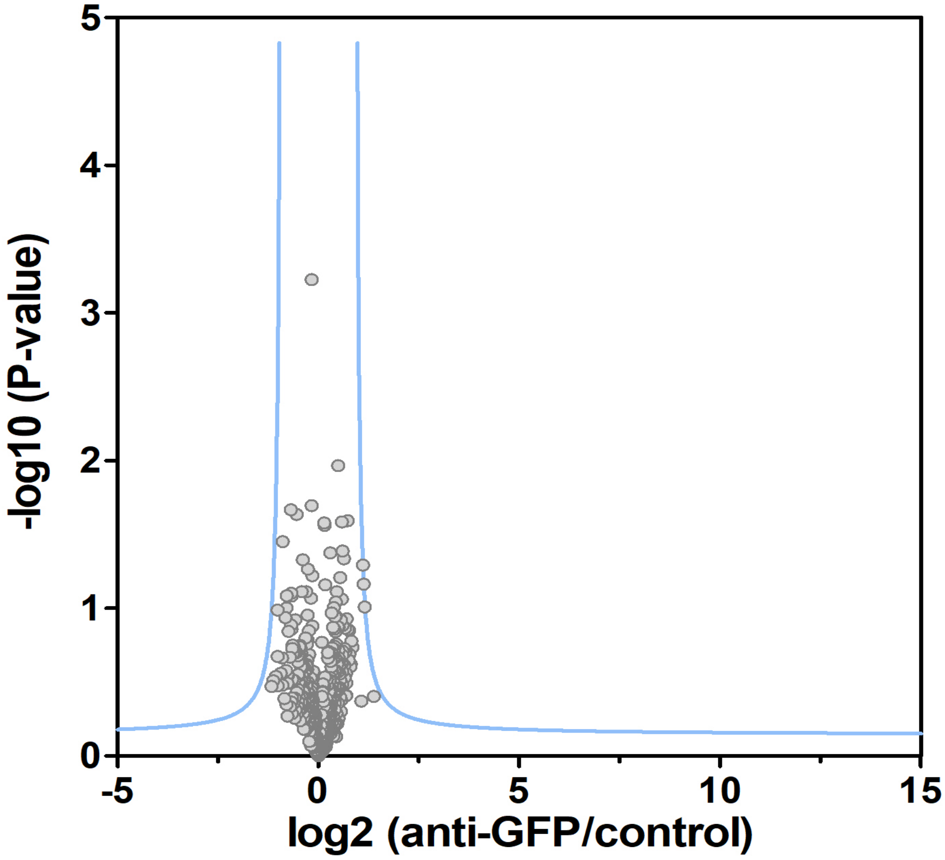

Fasimoye *et al*, Supplemental Figure 1

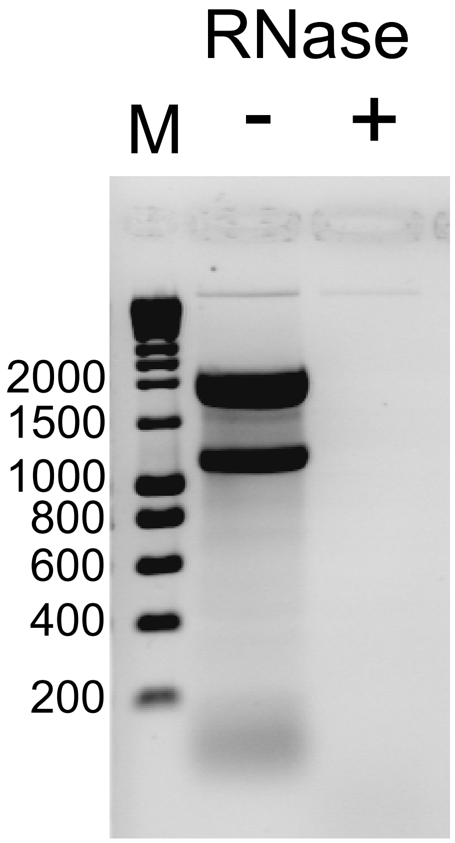

Fasimoye *et al,*
Supplemental Figure 2

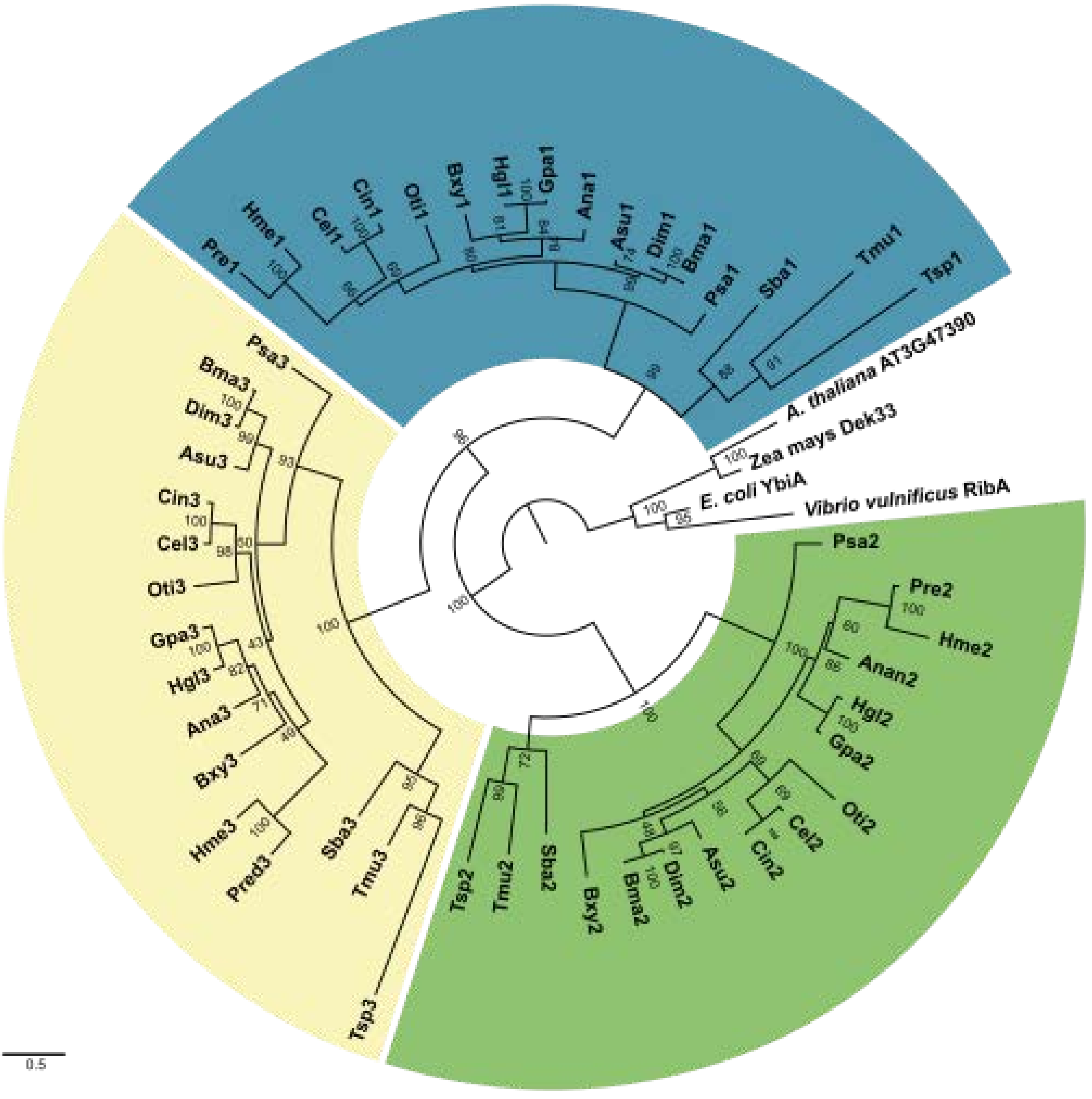

Fasimoye *et al*, Supplemental Figure 3

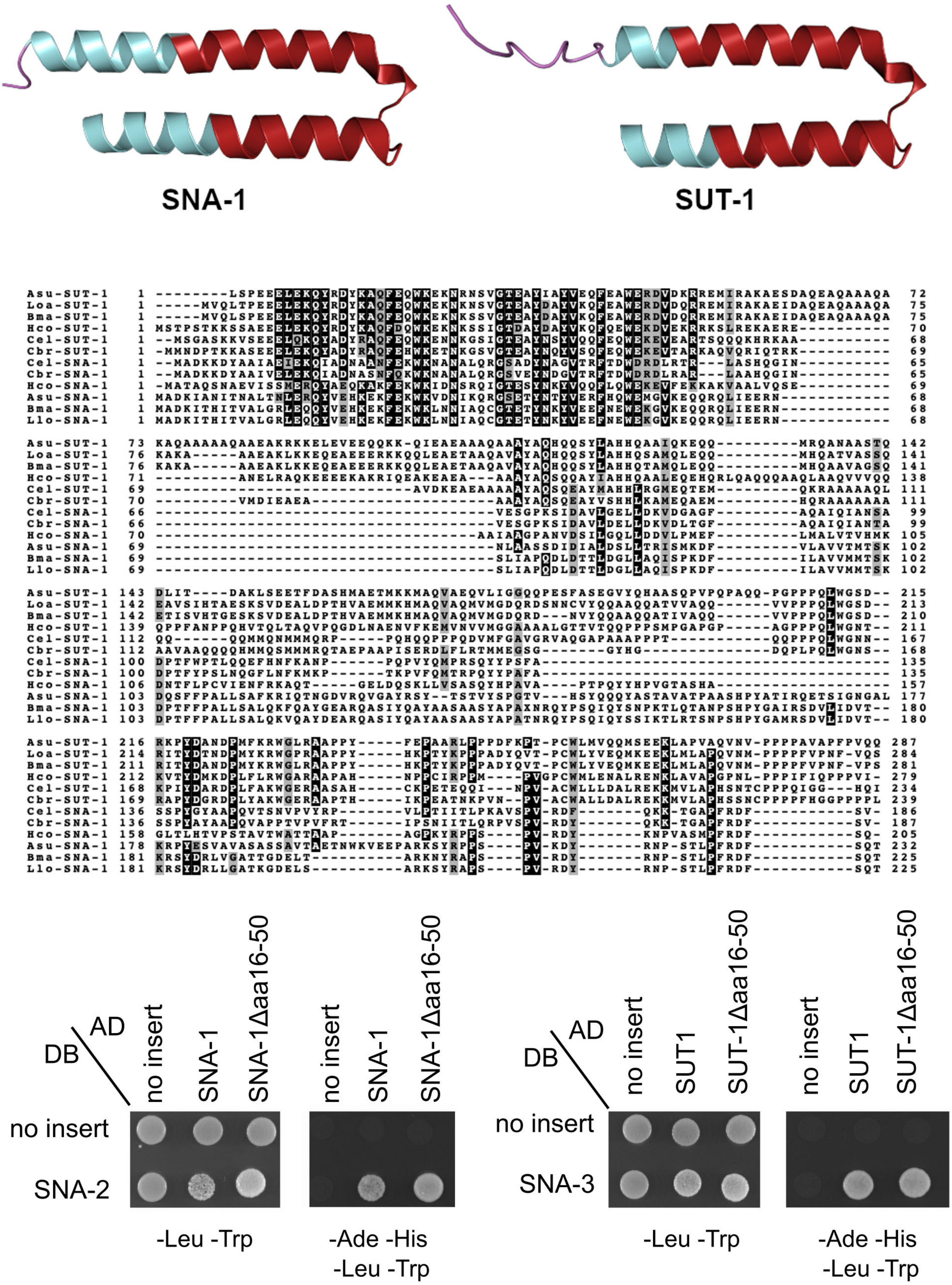

Fasimoye *et al*, Supplemental Figure 4

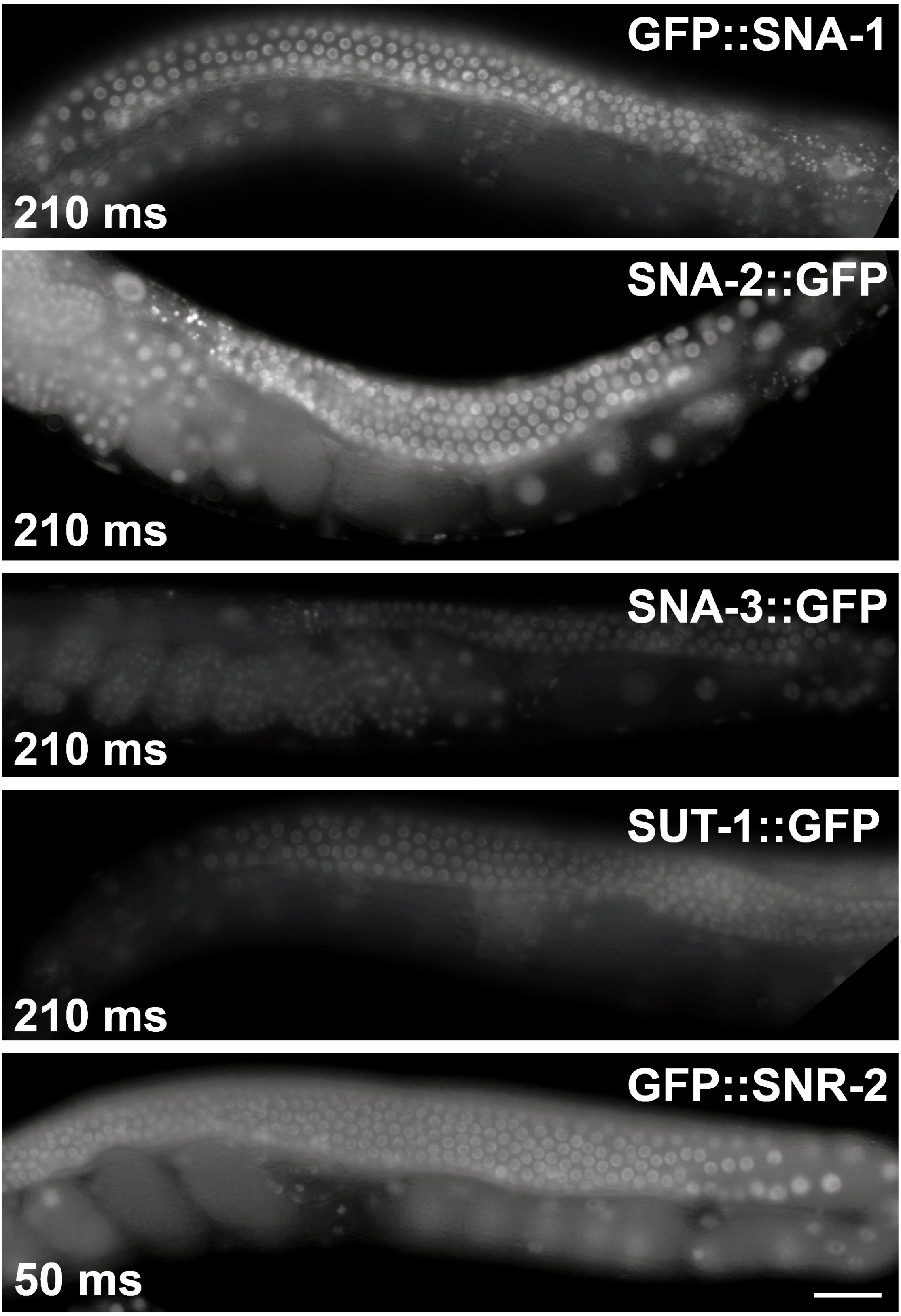

Fasimoye *et al*, Supplemental Figure 5

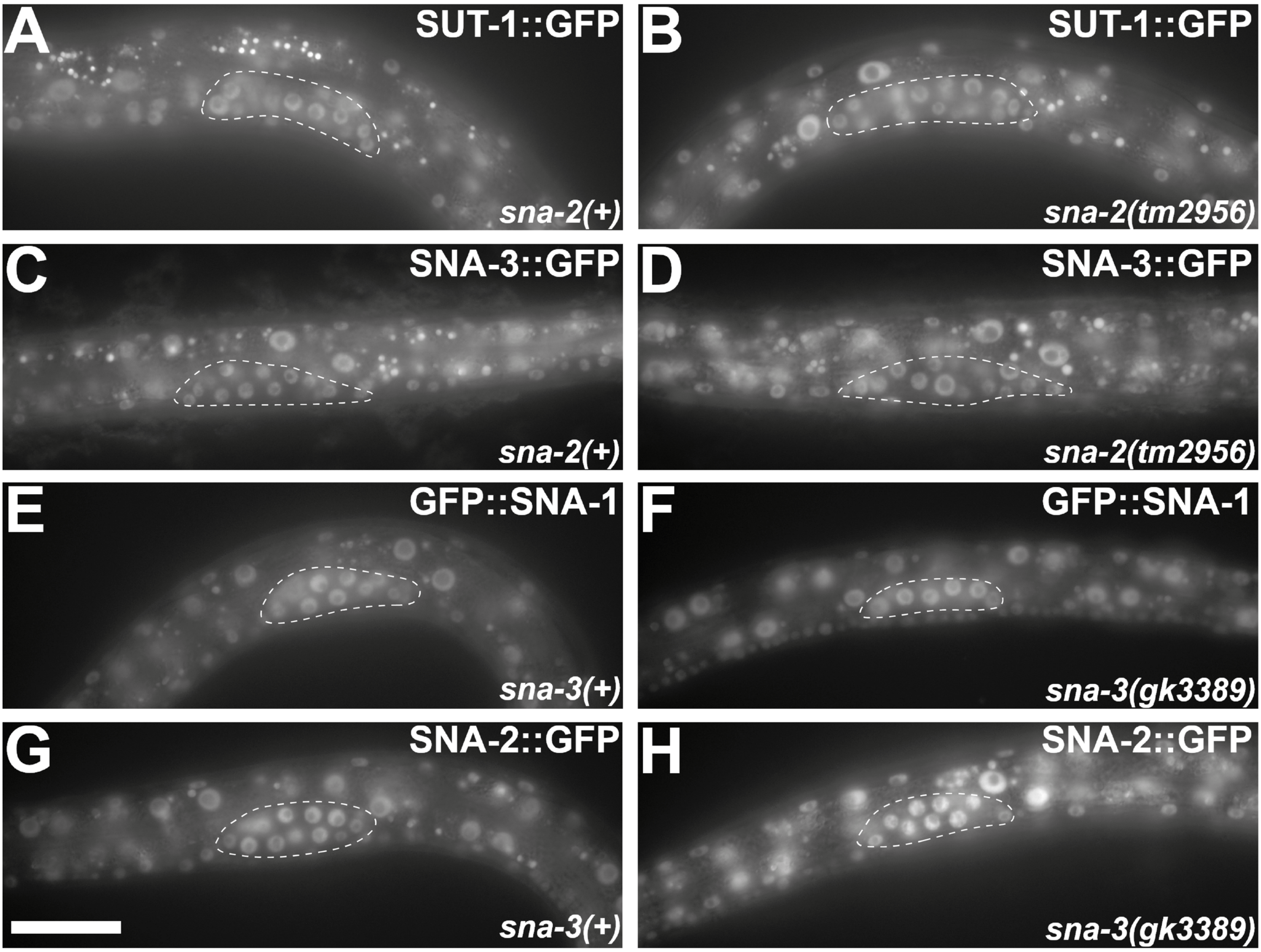

Fasimoye *et al*, Supplemental Figure 6

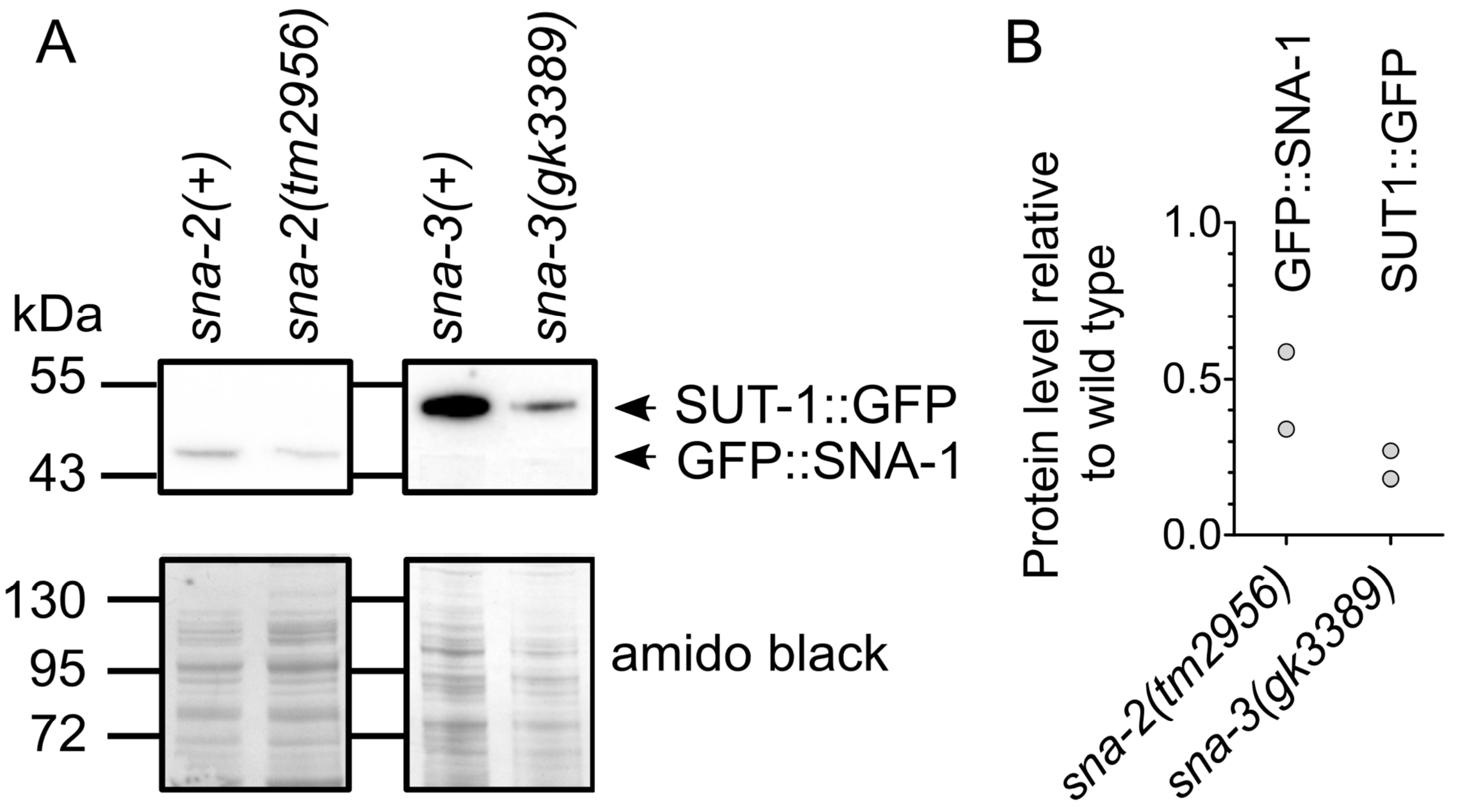

Fasimoye *et al,* Supplemental Figure 7
